# Genome-wide association reveals novel genomic loci controlling rice grain yield and its component traits under water-deficit stress during the reproductive stage

**DOI:** 10.1093/jxb/ery186

**Published:** 2018-05-15

**Authors:** Niteen N Kadam, Paul C Struik, Maria C Rebolledo, Xinyou Yin, S V Krishna Jagadish

**Affiliations:** 1 International Rice Research Institute, DAPO, Metro Manila, Philippines; 2 Centre for Crop Systems Analysis, Department of Plant Sciences, Wageningen University & Research, AK Wageningen, The Netherlands; 3 CIRAD, UMR AGAP, Montpellier, France. AGAP, Univ Montpellier, CIRAD, INRA, Montpellier SupAgro, Montpellier, France; 4 CIAT, Agrobiodiversity, AA, Cali, Colombia; 5 Department of Agronomy, Kansas State University, Manhattan, KS, USA

**Keywords:** *A priori* candidate genes, multi-locus analysis, *Oryza sativa*, reproductive-stage water-deficit stress, single-locus analysis, synchronized phenology

## Abstract

A diversity panel comprising of 296 indica rice genotypes was phenotyped under non-stress and water-deficit stress conditions during the reproductive stage in the 2013 and 2014 dry seasons (DSs) at IRRI, Philippines. We investigated the genotypic variability for grain yield, yield components, and related traits, and conducted genome-wide association studies (GWAS) using high-density 45K single nucleotide polymorphisms. We detected 38 loci in 2013 and 64 loci in 2014 for non-stress conditions and 69 loci in 2013 and 55 loci in 2014 for water-deficit stress. Desynchronized flowering time confounded grain yield and its components under water-deficit stress in the 2013 experiment. Statistically corrected grain yield and yield component values using days to flowering helped to detect 31 additional genetic loci for grain yield, its components, and the harvest index in 2013. There were few overlaps in the detected loci between years and treatments, and when compared with previous studies using the same panel, indicating the complexity of yield formation under stress. Nevertheless, our analyses provided important insights into the potential links between grain yield with seed set and assimilate partitioning. Our findings demonstrate the complex genetic architecture of yield formation and we propose exploring the genetic basis of less complex component traits as an alternative route for further yield enhancement.

## Teaser

Genetic analysis of grain yield and its components in a rice diversity panel identified genetic loci that regulate water deficit tolerance during the reproductive stage.

## Introduction

Rice (*Oryza sativa* L.) is a staple food crop for more than half the world’s population. Maintaining its high yield potential with sustained productivity is imperative for future food security. However, global climate change, with frequent episodes of abiotic stress (water deficit and heat stress), reduces the productivity of rice (Kadam *et al.*, 2014; Reynolds *et al.*, 2016), as rice is more sensitive to water deficit than other cereals (Kadam *et al.*, 2015). Nearly 20% of global rice production is affected by water deficit (Bouman *et al.*, 2005). Water deficit can occur at any time during the growing season, but stress occurring within the reproductive phase (i.e. from meiosis to flowering) causes the greatest grain yield losses (Liu *et al.*, 2006). The physiological effects of water deficit within the reproductive phase have been discussed in detail by Saini and Lalonde (1997), Saini *et al.* (1999), and Barnabás *et al.* (2008).

Increasing tolerance to water deficit has been considered as a major breeding target, although knowledge on phenotypic traits linked with stress tolerance is limited. Recent evidence in rice has demonstrated that progress can be made through direct selection of grain yield, as a criterion under reproductive-stage water deficit (Venuprasad *et al.*, 2007; Kumar *et al.*, 2014). Physiologically, grain yield is a very complex trait determined by different component traits (Slafer, 2003). Hence, exploring ideotype breeding based on selection for component traits is proposed as a complementary route for further yield improvement (Donald, 1968).

Revealing the genetic basis of grain yield and its component traits is essential for providing breeders with the tools for efficient development of stress-resilient cultivars. The genetic control of grain yield under reproductive-stage water deficit has been investigated extensively using linkage analysis of bi-parental crosses in rice. This approach has proven to be powerful in the detection of quantitative trait loci (QTLs) for grain yield and its components under stress (Lanceras *et al.*, 2004; Bernier *et al.*, 2007; Vikram *et al.*, 2011; Mishra *et al.*, 2013; Dixit *et al.*, 2014; Kumar *et al.*, 2014). A few of these QTLs regulating grain yield, for instance *qDTY*_*12.1*_, have been introgressed into elite cultivars to improve stress tolerance (Mishra *et al.*, 2013), but most of them are only based on a small fraction of the rice genetic diversity. Identifying the allelic variations exhibited in a large genetic diversity panel as a result of divergent selection pressure provides an obvious alternative that can have a greater potential in grain yield improvement under water deficit. These natural allelic variations have been identified in rice under non-stress conditions for grain yield and its component traits through genome-wide association studies (GWAS) (Agrama *et al.*, 2007; Borba *et al.*, 2010; Huang *et al.*, 2010, 2012; Zhao *et al.*, 2011; Begum *et al.*, 2015; Spindel *et al.*, 2015; Rebolledo *et al.*, 2016; Yano *et al.*, 2016). Yet, very few studies are available for reproductive-stage water-deficit conditions (Ma *et al.*, 2016; Pantalião *et al.*, 2016; Swamy *et al.*, 2017). This is partly due to the difficulty in implementing water deficit to coincide with reproductive stage under field conditions for a large diversity panel, which usually consists of genotypes having diverse phenology. Only the study of Ma *et al.* (2016) followed a staggered sowing to account for variation in flowering phenology under stress.

Our study aimed to (i) explore the natural variation in grain yield and yield component traits under non-stress and reproductive-stage water-deficit conditions; (ii) link the variation of these phenotypic traits with single nucleotide polymorphisms (SNPs) through GWAS; and (iii) identify the most likely underlying candidate genes in close proximity to the significant SNPs.

## Materials and methods

### Association mapping population

We used a rice panel consisting of a diverse set of 296 indica genotypes consisting of improved and traditional genotypes with (sub)tropical adaptation. This panel was assembled at the International Rice Research Institute (IRRI), Philippines for the Phenomics of Rice Adaptation and Yield potential (PRAY) project in the context of the Global Rice Phenotyping Network (http://ricephenonetwork.irri.org). Recent studies have reported GWAS analyses using this population for grain quality traits (Qiu *et al.*, 2015), salinity tolerance (Al-Tamimi *et al.*, 2016), panicle architecture (Rebolledo *et al.*, 2016), yield traits under varying planting densities (Kikuchi *et al.*, 2017), and root plasticity (Kadam *et al.*, 2017).

### Strategy to cope with variation in flowering phenology

**Fig. 1.**
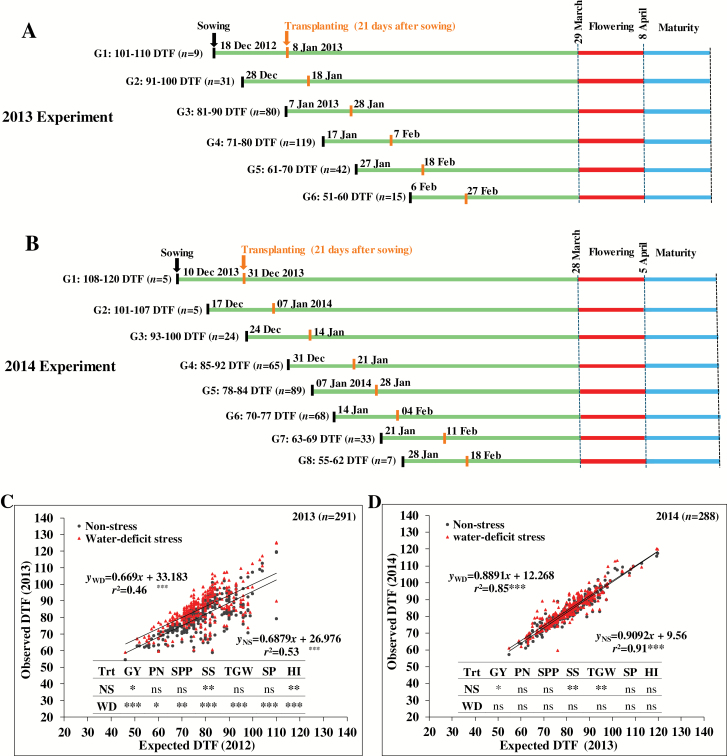
Schematic representation of the staggered sowing and transplanting approach to synchronize flowering time that was followed for screening of an indica rice diversity panel under reproductive-stage water-deficit stress in the dry seasons (DSs) of 2013 (A) and 2014 (B). Days to flowering (DTF) was 10 d between groups (G) in 2013 and 7 days in 2014 DS experiments. (C, D) The expected and observed DTF in non-stress (NS) and water-deficit stress (WD) in the 2013 (C) and 2014 (D) DS experiments. ANOVA results with the effect of DTF (as a covariate in mixed linear model) on grain yield and its key component traits are shown. GY, grain yield; HI, harvest index; *n*, number of genotypes; PN, panicles per m^2^; SP, spikelets per m^2^; SPP, spikelets per panicle; SS, seed set; TGW, thousand grain weight; Trt, treatments. Significance levels: *P<0.05, **P<0.01, ***P<0.001. To synchronize the flowering time, we used the 2012 wet season DTF data in the 2013 DS experiment (C). Similarly, for the 2014 DS experiment, we used DTF data from the 2013 DS experiment (D).

The PRAY panel was screened in non-stress and reproductive-stage water-deficit conditions under field experiments conducted at the upland farm of IRRI, Philippines (14°11′N, 121°15′E; elevation 21 m above sea level) in the 2013 and 2014 DSs. Seeds were sown from December of the preceding year to late January or early February of each year (Fig. 1). As expected, a strong genotypic variation in flowering phenology was observed that confounds the true water-deficit response (Fukai *et al.*, 1999) and inevitably induces bias with interpretation of genetic mapping outcomes (Pinto *et al.*, 2010; Kumar *et al.*, 2014). We followed staggered sowing in seedbeds and transplanting in main plots to synchronize flowering and thus minimize phenological differences under stress imposition (Fig. 1). Briefly, in the 2013 DS experiment, we divided 296 genotypes into six groups with a 10 d interval based on days to flowering data collected from a previous experiment conducted in the 2012 wet season (WS), our only source of flowering dates for this population grown at IRRI. While the expected range of flowering was 29 March to 8 April 2013 (Fig. 1A), we observed deviation in days to flowering in the 2013 DS experiment, where the staggered sowing was based on the 2012 WS data. Therefore, in the 2014 DS experiment, we regrouped the 296 genotypes into eight groups with a 7 d interval using 2013 DS flowering data to improve synchrony within the whole population. The expected date of flowering was 28 March to 5 April 2014 for these genotypes (Fig. 1B). In each year, the sowing date chosen for the stress treatment was the same as for the non-stress treatment of the same genotype.

### Crop management

The soil type of the upland farm at IRRI is Maahas clay loam, isohyperthermic mixed Typic Tropudalf. The experiments were laid out in a group block design with three replications for each genotype in both treatments (Supplementary Fig. S1 at *JXB* online). Seeds were first exposed to 50 °C for 3 d to break dormancy and then hand sown in a seedbed nursery. Twenty-one-day-old seedlings were transplanted (two seedlings per hill) for each genotype in four rows per replication. In both years, row distance was 0.2 m and row length was 2.4 m. The seeds of one genotype in 2013 and eight genotypes in 2014 germinated poorly and hence were excluded. In addition, four genotypes completed flowering and maturity before stress imposition in 2013 and were excluded. This resulted in final sets of 291 genotypes in 2013 and 288 genotypes in 2014, with three replications and two treatments totalling 1746 and 1728 plots in 2013 and 2014, respectively. A day before transplanting, 30 kg P ha^−1^ (as single superphosphate), 40 kg K ha^−1^ (as KCl), and 5 kg Zn ha^−1^ (as zinc sulfate heptahydrate) fertilizers were manually applied. Nitrogen fertilizer as urea was applied in three splits: 45 kg ha^−1^ before transplanting, 30 kg ha^−1^ at mid-tillering, and 45 kg ha^−1^ at panicle initiation. The IRRI standard management practices were followed to control weeds, insects, and diseases. In both years, all plots were maintained like irrigated lowlands with ~5 cm standing water until maturity except for the water-deficit plots during the stress period (see below).

### Reproductive stage water-deficit stress imposition

There was variation in synchronizing days to flowering among rice genotypes in 2013, resulting in deviation from our expected flowering window (29 March to 8 April). In rice, the reproductive stage ranges between 19 and 25 d, starting at panicle initiation and ending with flowering (Moldenhauer *et al.*, 2013). Therefore, before imposing stress, we manually dissected the main tillers of the middle two plants of border rows from water-deficit plots for all the genotypes, primarily to check the reproductive-stage development. Stress was imposed on 23 March 2013 when the majority of genotypes reached the agronomic panicle initiation stage, by draining water out from the field. The stress continued for 14 d until 5 April 2013. In the 2014 experiment, the synchronization was more precise with expected dates of flowering occurring between 28 March and 5 April, as predicted. The same dissection approach as in 2013 was followed and stress was imposed on 26 March 2014 and continued for 14 d until 8 April.

To quantify the stress intensity, 26 tensiometers were installed randomly across the entire stress field at 30 cm depth in each season. A polythene sheet was inserted at 2 m depth by digging a deep and narrow trench in between stress and non-stress fields to prevent water seepage during the stress period from the adjacent non-stress field. In addition, the stress and the non-stress plots were separated by a distance of 2.3 m (Supplementary Fig. S1). The intensity of stress was higher in 2014 than in 2013 (Supplementary Fig. S2A). There was no rainfall during the peak stress period in both seasons, except for rainfall during the first day of the stress period in 2013 (Supplementary Fig. S2B). Higher stress intensity in 2014 compared with 2013 could be due to higher maximum temperature and higher vapour-pressure deficit (Supplementary Fig. S3B, D), leading to quicker loss of soil moisture in 2014. A weather station was placed between the non-stress and water-deficit plots (see Supplementary Fig. S1). Detailed weather data are given in Supplementary Fig. S3.

### Observations

At maturity, plants of 16 hills from the middle two rows, i.e. 0.64 m^2^ plot area (excluding the border rows) were harvested to assess yield (14% moisture), its components, and related traits in both experiments, following Shi *et al.* (2016). Days to flowering was assessed as the interval between the date of sowing and the date when panicles of 50% of plants per plot were fully exerted. Days to maturity was assessed as the interval between the flowering date and date when panicles on most plants in a plot turned yellow and ready for harvest. Plant height was measured from the base of the root–shoot junction to the tip of the flag leaf, which was manually straightened to be aligned with the culm. Non-grain dry weight was assessed as the sum of leaf, stem and rachis dry weight. The total aboveground dry weight was the sum of non-grain and grain dry weight. Harvest index was the ratio of grain dry weight to total aboveground dry weight.

### Statistical analysis of phenotypic data

#### Analysis of variance

A combined linear mixed model based analysis of variance (ANOVA) was performed to test the effect of genotype (G), treatment (T), and year (Y) with their interactions using the following model in Genstat V17.1:

$$Y_{ijkl}=µ+G_{i}+T_{j}+Y_{k}+R_{l}[\left(T_{j}\left(Y_{k}\right)\right]+{\left(G\times T\times Y\right)}_{ijk}+E_{ijkl}$$

where *Y*_*ijkl*_ is the phenotypic trait value recorded in a plot, µ is the overall mean, *G*_*i*_ is the effect of the *i*th genotype, *T*_*j*_ is the effect of the *j*th treatment, *Y*_*k*_ is the effect of the *k*th year, *R*_*l*_[*T*_*j*_(*Y*_*k*_)] is the effect of the *l*th replication within the *j*th treatment of the *k*th year, (*G*×*T*×*Y*)_*ijk*_ is the effect of three-way interaction between the *i*th genotype, the *j*th treatment and the *k*th year, and *E*_*ijkl*_ is the error. Apart from the three-way interaction, we also consider two-way interactions of main factors in all possible combinations.

#### Linear mixed model to estimate best linear unbiased estimators

We estimated the best linear unbiased estimators (BLUEs) of phenotypic traits for an individual genotype across years and treatments separately. The following linear mixed model was used in Genstat V17.1 to estimate the BLUEs separately in non-stress and stress conditions across years, using genotypes as a fixed effect and replications as a random effect,

$$Y_{ij}=µ+G_{i}+R_{j}+E_{ij}$$

where *Y*_*ij*_ is the phenotypic trait value recorded in a plot, µ is the overall mean, *G*_*i*_ is the effect of the *i*th genotype, *R*_*j*_ is the effect of the *j*th replication, and *E*_*ij*_ is the error.

Days to flowering had a strong confounding effect on grain yield and its components under stress, particularly in 2013 (Fig. 1C). Therefore, we performed the linear mixed model-based ANOVA using the above equation with days to flowering as covariate. When the effect of days to flowering was significant on phenotypic traits, corrected BLUEs of trait values were estimated in stress treatments.

#### *Principal component analysis*, *trait correlation and multiple regression analysis*

A multivariate principal component analysis (PCA) was performed in XLSTAT across years and treatments. The chart.Correlation() function within the R package ‘Performance Analytics’ was used to generate the correlation scatter plot. The lm() function in R was used for multiple linear regression analysis of grain yield with its component and related traits.

#### Heritability estimates

Broad-sense heritability (*H*^2^), capturing the proportion of phenotypic variance explained by genetic factors that is due to dominance, epistatic, and additive effects, was calculated across years and treatments separately using the below equation:

$$H^{2}=\frac{\sigma_{\text{G}}^{2}}{\sigma_{\text{G}}^{2}+\frac{\sigma_{\text{E}}^{2}}{r}}$$

where σ^2^_G_ and σ^2^_E_ are the genotypic and residual variances, respectively, and *r* is the number of replications. The restricted maximum likelihood estimate was used to calculate the variance components in Genstat V17.1. The narrow-sense heritability (*h*^2^), capturing the proportion of total phenotypic variance explained by the additive genetic variance, was estimated using the equation in Genomic Association and Prediction Integrated Tool (GAPIT) function:

$$h^{2}=\frac{\sigma_{\text{a}}^{2}}{\sigma_{\text{a }}^{2}+\sigma_{\text{e}}^{2}}$$

where σ^2^_a_ is the additive genetic variance and σ^2^_e_ is the residual variance.

### Genetic analysis of marker-trait associations

Two hundred and ninety-one genotypes in 2013 and 288 genotypes in 2014 had complete phenotypic data. However, 20 genotypes were missing from the 45699 (46K) SNPs dataset resulting in 271 genotypes in 2013 and 268 in 2014, used for GWAS analysis. The detailed genotype-by-sequencing protocol of SNP genotyping, population structure, and linkage disequilibrium (LD) for this population is explained in Kadam *et al.* (2017). The GWAS was performed on a set of 271 (2013) and 268 (2014) genotypes separately, with 267 genotypes being common across both years. Two GWAS methods were used to test the marker–trait associations: single-locus and multi-locus analysis.

Single-locus analysis is a one-dimensional scan, typically identifying associations between single markers and traits. We performed this analysis using a compressed mixed-linear model (CMLM; Zhang *et al.*, 2010) in GAPIT (Lipka *et al.*, 2012). In the mixed model, we included population structure and family kinship (family relatedness), which were calculated by the GAPIT function using SNPs with ≥0.05 minor allele frequency (MAF).

$$Y=X_{\text{α}}+Q_{\text{β}}+K_{\text{μ}}+e$$

where *Y* represents the vector of phenotype, *X* represents the vector of SNPs, *Q* is the PCA matrix and *K* is the relative kinship matrix. *X*α and *Q*β are the fixed effects, and *K*μ and *e* represent random effects. The *Q* and *K* matrices help to reduce the spurious false positive associations. Correction for population structure (*Q*) substantially reduces the false positives but it sometimes eliminates true positive associations due to overcorrection. Therefore, the optimal number of principal components was estimated for each trait before incorporating them for CMLM tests, based on the forward model selection method using the Bayesian information criterion. This method helps to control both false-positive and -negative associations more effectively although it cannot eliminate both completely. We used a lower suggestive threshold probability *P*-value 1.0 × 10^−4^ (−log_10_*P*=4) and superior Bonferroni corrected threshold as an upper limit (2013: −log_10_(0.05/45437)=6; 2014: −log_10_(0.05/45414)=6) to detect significant associations.

The single-locus analysis corrects the confounding effects of population structure and family kinship but does not consider the confounding effect of causal loci. The multi-locus GWAS is a method that corrects not only the confounding effects of population structure and family kinship but also the confounding and/or interaction effects of causal loci present in the genome due to LD (Segura *et al.*, 2012). We performed the multi-locus GWAS using a modified version of the multi-locus mixed linear model (MLMM) in R (R script for mlmm.cof.r available at https://cynin.gmi.oeaw.ac.at/home/resources/mlmm). We ran the complete model as recommended with stepwise forward inclusion of the strongest significant markers (lower *P*-value) and stepwise backward elimination of the last forward model (that is, least significant markers). Significant markers were selected based on the criteria explained by Kadam *et al.* (2017). Briefly, in the first step (like single-locus GWAS without any marker as a cofactor), we manually checked the *P*-value of SNPs before including them as a cofactor in the model. Then we continued adding markers to the model as cofactors based on cut-off threshold *P*-value≤1.00 × 10^−4^. Once no significant loci appeared below the threshold *P*-value, the model was stopped. All the significant cofactors identified were considered as significant loci.

### Selecting *a priori* candidate genes underlying the genetic loci

The detailed protocol to select *a priori* candidate genes near to significant SNPs was followed as explained in Kadam *et al.* (2017).

## Results

### The flowering time was sensitive to seasonal climate variations

The flowering time synchronization approach was followed to reduce the confounding effect of flowering time differences of rice genotypes on grain yield and its components (those measured in this study) and related traits under stress (Fig. 1A, B). However, we witnessed deviation of our observed days to flowering from expected days (*r*^2^=0.53 in non-stress and *r*^2^=0.46 in stress conditions; Fig. 1C) in 2013. As rice flowering time is regulated by internal genetic cues and external stimuli such as photoperiod and temperature (Yin *et al.*, 1997), such deviations were expected, since the synchronization in 2013 was based on 2012 WS pre-experiment data due to lack of DS data. Many genotypes exhibited photothermal sensitivity across wet and dry seasons. Therefore, some genotypes experienced stress during the flowering period (31%), whereas others experienced stress either before (60%) or immediately after flowering (8%). In 2014, we restructured the synchronization based on 2013 DS data. This resulted in better synchronization with only small deviation observed from expected days to flowering (*r*^2^=0.91 in non-stress and *r*^2^=0.85 in stress conditions; Fig. 1D). Further, to test the effect of days to flowering, we performed the analysis with days to flowering as a covariate in the mixed model. The moderate to strong significant effect of days to flowering on yield, its components, and harvest index were detected in 2013 stress, most likely due to desynchronized flowering time. Conversely, the improved flowering synchronization caused no significant effect in 2014 stress. The marginal (*P*<0.05) to moderate (*P*<0.01) effect of days to flowering on yield, seed set, and harvest index was detected in both years under non-stress (Fig. 1C, D). This could be due to the pleiotropic effect of flowering genes on panicle development (Crowell *et al.*, 2016), a key determinant of rice grain yield.

### Genotype effects and genotype-by-environment interactions accounted for variations in phenotypic traits

**Fig. 2.**
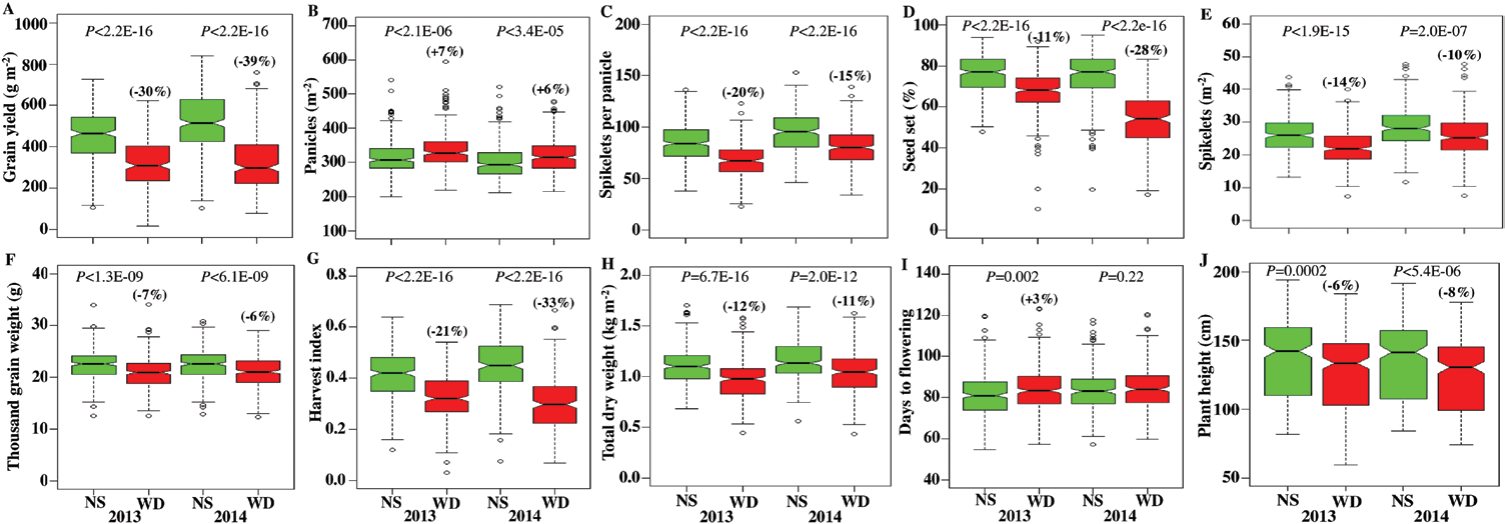
Box-plot showing phenotypic distribution of grain yield and its components and related traits in non-stress (NS) and water-deficit stress (WD) during 2013 (*n*=271) and 2014 (*n*=268). Two-sample *t*-test *P*-value shows the significant difference between grain yield (A), its components (B–F), and related traits (G–J) in NS and WD conditions. *n*, number of genotypes. Inside boxplot, the bold line represents the median, box edges represent upper and lower quantiles, and whiskers are 1.5× the quantile of the data. Outliers are shown as open circles. Values in parentheses represent the significant percentage change (increase (+) or decrease (−)) in WD over NS conditions. Days to maturity across treatments in 2013 and data for non-grain tissue dry weight across treatments and years are given in Supplementary Table 1. The values of phenotypic traits given in the box-plot under 2013 water deficit are the original, not corrected for days to flowering to account variation in flowering synchronization.

A combined mixed model ANOVA across years was carried out to divide the variation in genotype, treatment and year components and their interactions (Table 1). The variation in grain yield, its components, and other related traits differed significantly between genotypes (G; *P*<0.001), treatments (T; *P*<0.001) and years (Y; *P*<0.01 to *P*<0.001). Further, the yield, its component, and related traits of each genotype responded differently to treatment (G×T; *P*<0.001) and year (G×Y; *P*<0.001). The detailed descriptive statistics of these traits are given in Supplementary Table S1. The traits showed different distributions in non-stress and stress conditions for both years (Fig. 2). Yield ranged from 106.3 to 727.0 g m^−2^ in non-stress, and from 16.7 to 622.6 g m^−2^ under stress in 2013, and from 102.8 to 839.7 g m^−2^ in non-stress, and from 78.1 to 761.1 g m^−2^ under stress conditions in 2014. Across all observations, *H*^2^ and *h*^*2*^ estimates ranged from 0.73 to 0.99 and from 0.27 to 0.94, respectively, in 2013; and from 0.62 to 0.99 and from 0.69 to 0.93, respectively, in 2014 (Supplementary Table S1). The greater reduction of yield, seed set, and harvest index under stress in 2014 was due to higher stress intensity during 2014 (−64 kPa) compared with 2013 (−46 kPa), driven by higher vapour-pressure deficit (Supplementary Figs S2A, S3D). However, a higher reduction of spikelets per panicle and spikelets per m^2^ despite lower stress intensity was observed during 2013 than during 2014 (Fig. 2C, E). This could be due to variation in flowering time synchronization with more genotypes experiencing stress before flowering in 2013 than in 2014. These results clearly illustrate that stress affects the number of spikelets per m^2^ when imposed before flowering, but spikelet fertility when imposed during flowering (Lanceras *et al.*, 2004), as shown in Fig. 2C, E. The days to flowering differed significantly (*P*=0.002) between non-stress and stress in 2013, but not (*P*>0.05) in 2014 (Fig. 2I).

**Table 1.** Analysis of variance (ANOVA) in 2013 and 2014 dry season experiments of three groups of traits: grain yield, yield components, and other related traits

Trait	Unit	G	T	Y	G×T	G×Y	T×Y	G×T×Y
Grain yield	g m^−2^	***	***	***	***	***	***	**
Grain yield component traits								
Panicles per m^2^	m^−2^	***	***	***	***	***	ns	***
Spikelets per panicle	—	***	***	***	***	***	ns	
Seed set	%	***	***	***	***	***	***	***
Thousand grain weight	g	***	***	**	***	***	ns	***
Spikelets per m^2^ (×10^3^)	m^−2^	***	***	***	***	***	***	
Other related traits								
Harvest index	—	***	***	***	***	***	***	***
Total dry weight	kg m^−2^	***	***	***	***	***	ns	ns
Non-grain dry weight	kg m^−2^	***	***	***	***	***	***	***
Plant height	cm	***	***	***	***	***	*	ns
Days to flowering	—	***	***	***	***	***	***	***
Days to maturity	—	—	—	—	—	—	—	—

Spikelets per m^2^ is not an independent yield component but is the product of panicles per m^2^ and spikelets per panicle. G, genotype; T, treatment; Y, year. Significance level: *P<0.05, **P<0.01, ***P<0.001; ns, non-significant.

**Fig. 3.**
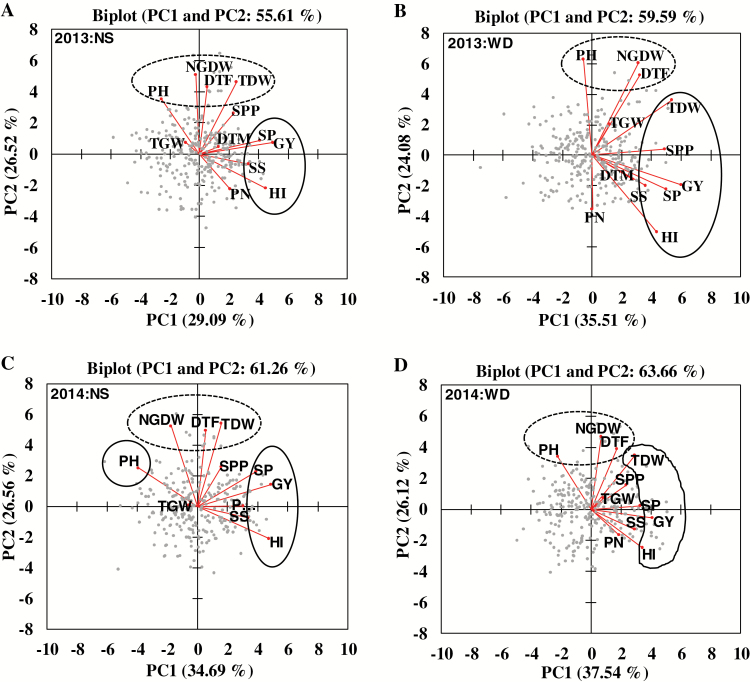
The principal component analysis of grain yield, its components, and related traits with first two principal components (PC1 and PC2) in non-stress (NS) (A, C) and water-deficit stress (WD) (B, D) during 2013 (A, B) and 2014 (C, D) DS. The traits marked inside the solid circle/ellipses contributed more to the variation explained by PC1 and those marked inside the dashed ellipses to PC2. DTF, days to flowering; DTM, days to maturity; GY, grain yield; HI, harvest index; NGDW, non-grain dry weight; PH, plant height; PN, panicles per m^2^; SP, spikelets per m^2^; SPP, spikelets per panicle; SS, seed set; TDW, total dry weight; TGW, thousand grain weight.

The first two principal components cumulatively explained >55% in 2013 and >61% in 2014 of the total phenotypic variation across treatments (Fig. 3). The genotypic variation in the first PC was mostly explained by yield, harvest index and spikelets per m^2^ in non-stress and yield, harvest index, spikelets per m^2^ and total dry weight under stress in 2013 and 2014. The genotypic variation in the second PC was explained by non-grain dry weight, days to flowering, and total dry weight under non-stress, and plant height, non-grain dry weight, and days to flowering under stress in 2013 and 2014. In addition, the principal component variations for the phenotypic traits differed in response to treatment and year (Fig. 3). This further confirms the strong G×T and G×Y interactions.

### Phenotypic trait correlations and contribution of component traits to grain yield

Grain yield was significantly (*P*<0.05) correlated with most of its components and related traits across treatments and years (Supplementary Figs S4, S5). However, non-significant (*P*>0.05) correlations of yield were found with thousand grain weight and non-grain dry weight in non-stress, and with panicle number in 2013 stress. Yield was not significantly (*P*>0.05) correlated with non-grain dry weight across treatments in 2014. The correlation of yield with spikelets per panicle was higher in stress (2013: *r*=0.73; 2014: *r*=0.46) than in non-stress conditions (2013: *r*=0.40; 2014: *r*=0.36) in both years, and the increase was stronger in 2013. Similarly, the correlation between yield and seed set increased from 0.62 in non-stress to 0.75 in stress conditions in 2014. The increased correlation of yield with spikelets per panicle in 2013 and with seed set in 2014 in stress conditions reflects the effect of variation in days to flowering synchronization. The correlation of yield with days to flowering was higher under stress (*r*=0.29) than under non-stress conditions (*r*=0.16) in 2013, but was almost the same (*r*=0.30) for both treatments in 2014.

We also tested the relative contribution of each component and related trait to yield through multiple linear regression. All the components and related traits significantly contributed to yield except for plant height and days to flowering in non-stress in 2013 and days to flowering in stress conditions during 2014 (Supplementary Table S2).

### Treatment and year specific genetic loci for phenotypic traits

Grain yield and its components and related traits followed a normal distribution (Supplementary Figs S4, S5), indicating the quantitative pattern suitable for genetic analysis. A summary of GWAS results using single-locus and multi-locus analysis methods is given in Table 2. The detailed results are in Supplementary Tables S3–S6. In total, we identified 38 significant loci in non-stress conditions, and 69 loci in stress conditions during 2013, and 64 significant loci in non-stress conditions, and 55 loci in stress conditions during 2014. Most loci were specific across treatments within years and within treatments across the years. Nevertheless, we also detected 14 common loci (nine in 2013 and five in 2014) across treatments and eight common loci within treatments (six in non-stress and two in stress conditions) across years for the same components and related traits (Supplementary Table S7).

**Table 2.** Summary of genetic loci detected in 2013 and 2014 under non-stress (NS) and water-deficit stress (WD) conditions for three groups of traits: grain yield, yield components, and other related traits

Traits	2013			2014	
	NS	WD	WD^*a*^	NS	WD
Grain yield	2	4	2	6	5
Grain yield component traits					
Panicles per m^2^	6	12	7	9	3
Spikelets per panicle	5	9	6	2	na
Seed set	3	7	7	8	11
Thousand grain weight	3	4	na	6	8
Spikelets per m^2^	1	4	1	4	3
Subtotal	18	36	21	29	25
Other related traits					
Harvest index	6	7	8	4	2
Total dry weight	3	2	—	4	11
Non-grain dry weight	3	3	—	5	2
Plant height	2	6	—	6	4
Days to flowering	3	11	—	10	6
Days to maturity	1	na	—	—	—
Subtotal	18	29	8	29	25
Total	38	69	31	64	55

na, no marker trait association analysis performed

^*a*^ Marker-trait associations detected for corrected trait values in water-deficit stress (see the text for the correction method).

### Genetic analysis after correcting for days to flowering under stress conditions in 2013

**Fig. 4.**
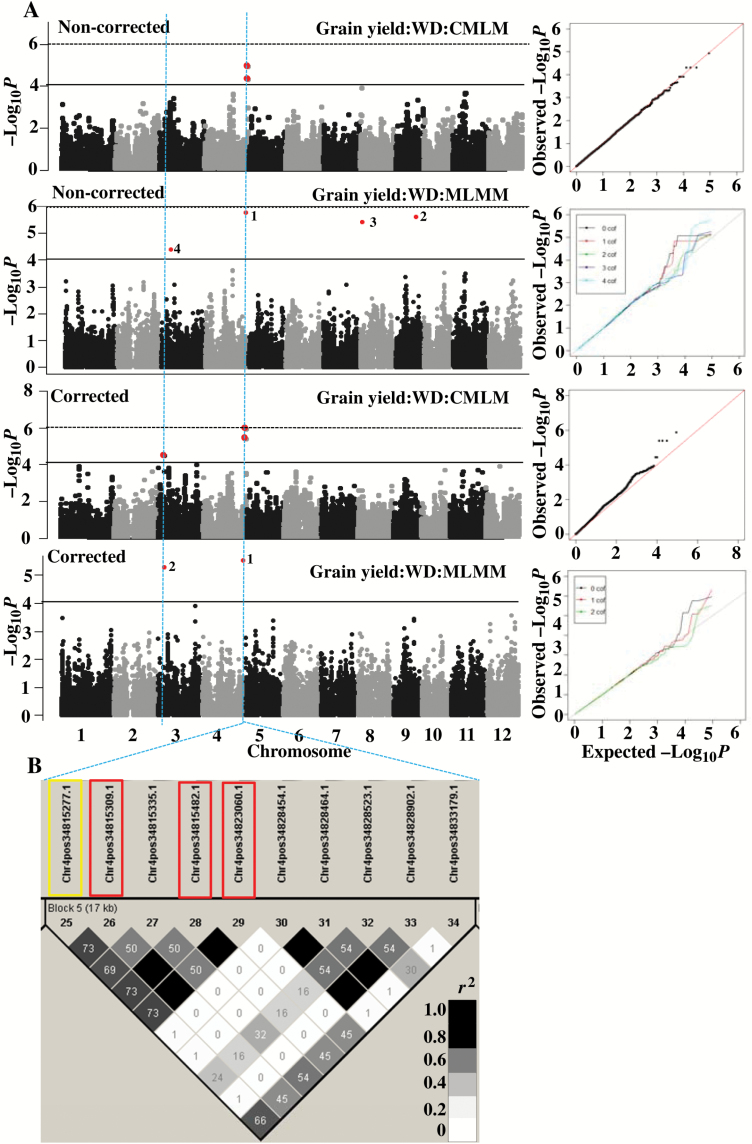
(A) GWAS results (Manhattan and quantile–quantile plot) detected through single-locus compressed mixed linear model (CMLM) and multi-locus mixed model (MLMM) for non-corrected and corrected (using days to flowering as covariate) grain yield in 2013 water-deficit stress (WD) conditions. Significant SNPs in the Manhattan plot of MLMM are numbered according to the order in which they were included as a cofactor in the regression model. (B) Identified LD block (17 kb) based on *r*^2^ value between SNPs on chromosome 4 and the colour intensity of the box on the LD plot corresponds with *r*^2^ (multiplied by 100) according to legend. Significant SNP marked by first rectangle was detected by CMLM and MLMM and the next three rectangles only by CMLM approach.

Flowering time synchronization was strongly confounding the grain yield and its component traits in 2013 stress conditions (Fig. 1C, D). We corrected for yield, yield components, and other related traits (only harvest index in this group) using days to flowering as a covariate in the mixed model. The single and multi-locus analysis of corrected trait values identified 31 additional loci using similar threshold *P*-values as mentioned earlier (Table 2; Supplementary Table S8). Most genetic loci detected for non-corrected traits disappeared when corrected trait values were subjected to GWAS analysis. This suggests that the trait variations associated with these loci were mostly explained by variation in days to flowering. Only five genetic loci (one on chromosome 4 for yield (Q9); one on chromosome 12 for spikelets per m^2^ (141599) and three loci on chromosome 11 for harvest index (10627944, 10131062, 10329677) were common to corrected and non-corrected trait values (Supplementary Tables S4 and S8). The common (corrected *vs* non-corrected) loci detected for yield (Q9; Table 3; Fig. 4) and harvest index (Supplementary Fig. S6; Supplementary Tables S4, S8) recorded lower *P*-values for corrected than the non-corrected trait value through single locus analysis. Despite correction, the novel locus Q10 on chromosome 3 for corrected yield, seed set, and harvest index overlapped with days to flowering (Table 3). In summary, statistical correction helped to explain the confounding effect of days to flowering and to some extent helped to eliminate its effect on yield under water deficit. Unless otherwise mentioned, all the mapping results discussed in the following sections were for the corrected trait loci under 2013 stress.

**Table 3.** GWAS results for final set of genetic loci detected for grain yield in non-stress and water-deficit stress conditions during 2013 and 2014. Detailed GWAS results for yield components and related traits across treatments and years are given in Supplementary Tables S3–S6 and S8

Treatment	Year (mean grain yield (g m^−2^))	Locus name	SNP pos^*a*^	Chr^*b*^	Allele	MAF^*c*^	*P*_CMLM_^*d*^	*P*_MLMM_^*e*^	AE^*f*^ (g m^−2^)	LD block^*g*^		Size (kb)	Known genes^*h*^
										Start	End		
Non-stress	2013 (451.1)	Q1	10101900	11	C:G	0.336	2.72 × 10^−5^	—	30.13	10101900	10173685	71	2
		**Q2**	**30523925**	**2**	**G:A**	**0.070**	**—**	**5.78 × 10** ^**−8**^	**−175.90**	**30397910**	**30541202**	**143**	**16**
	2014 (521.9)	Q3	13199901	12	C:T	0.468	6.84 × 10^−5^	—	30.04	12917853	13298195	380	8
		Q4	26796595	3^*k*^	C:T	0.097	9.91 × 10^−5^	2.20 × 10^−6^	−46.18	26756997	26978105	221	9
		Q5	29142398	2^*l*^	C:A	0.179	—	4.19 × 10^−5^	13.98	29122557	29261158	138	9
		Q6	19367031	10^*l*^	T:G	0.466	—	1.75 × 10^−6^	74.08	19280939	19474522	193	21
		Q7	5105627	12^*l*^	A:C	0.078	—	3.03 × 10^−5^	−186.24	5101105	5390949	289	12
		Q8	42643337	1^*l*^	A:G	0.347	—	6.58 × 10^−5^	−97.80	42587683	42643699	56	9
Water deficit	2013 (317.3)	Q9^*i*^	34815277	4	C:T	0.074	1.17 × 10^−5^	1.77 × 10^−6^	−81.29	34815277	34833179	17	5
		—	—	—	—	—	***1.29 × 10*** ^***−6***^	***3.05 × 10*** ^***−6***^					
		Q10^*j*^	5113428	3^*k*^	T:C	0.424	3.55 × 10^−5^	5.17 × 10^−6^	−40.61	5 021 158	5167439.00	146	13
	2014 (319.5)	Q11	6934188	3^*k*^	A:G	0.397	8.26 × 10^−5^	8.73 × 10^−6^	31.47	6 908 684	7020707	112	7
		Q12	42144827	1^*l*^	T:C	0.366	—	1.86 × 10^−7^	6.10	42 123 552	42144993	21	2
		Q13	16038003	10^*l*^	T:C	0.358	—	5.46 × 10^−6^	−49.86	16 024 382	16110372	85	6
		Q14	23005301	11^*l*^	G:A	0.276	—	1.12 × 10^−5^	−23.54	22 976 390	23005386	28	0
		Q15	27115652	11^*l*^	G:A	0.075	—	4.92 × 10^−5^	33.65	27 115 609	27123090	7	1

^*a*^ Single nucleotide polymorphism (SNP) position.

^*b*^ Chromosome.

^*c*^ Minor allele frequency (MAF).

^*d*^ *P*-value of single-locus compressed mixed linear model (CMLM).

^*e*^ *P*-value of multi-locus mixed model (MLMM).

^*f*^ Allelic effect with respect to minor allele=(average traits value of genotypes carrying minor allele−average traits value of genotypes carrying major allele).

^*g*^ Linkage disequilibrium block.

^*h*^ Total number of known characterized genes in LD block.

^*i*^ Genetic locus detected for non-corrected and corrected grain yield value.

^*j*^ Genetic locus detected for corrected grain yield value and coinciding with days to flowering.

^*k*^ Genetic locus detected through CMLM and MLMM methods.

^*l*^ Genetic locus detected through MLMM method only.

All the unmarked loci were detected through CMLM method. The italic *P*-value is for corrected grain yield value. The genetic locus marked in bold (Q2) overlaps with panicle weight (equivalent to grain yield) from Kikuchi *et al.* (2017) (Supplementary Table S10).

### Eight grain-yield loci revealed small to medium allelic effect in non-stress conditions

We identified two (Q1 and Q2) and six (Q3–Q8) loci for grain yield in 2013 and 2014, respectively (Table 3). There were no common loci across years, most likely due to significant variations in temperature (minimum and maximum) and vapour-pressure deficit (VPD; Supplementary Fig. S3). These loci had a positive or negative effect (small to medium) on yield with regard to its minor allele (allele refers to the 0.05 frequency in the studied population). In 2013, the minor allele of Q1 had a positive effect on yield. Conversely, the minor allele of Q2 had a negative effect on yield. In 2014, the minor allele of Q3, Q5, and Q6 had a positive effect, while the minor allele of Q4, Q7, and Q8 had a negative effect on yield (Table 3).

Eighteen and sixty-eight *a priori* (known or characterized) candidate genes were harboured within the expected LD block by Q1 and Q2 in 2013, and Q3–Q8 in 2014, respectively. Interestingly eight *a priori* candidate genes were identified (Supplementary Table S9). Q1 was close to *Os*PTR2 (6 and 31 kb; two copies in LD block). The rice homologue of this gene, *short panicle 1* (*Os*PTR2), regulates panicle and grain size and nitrate transport (Li *et al.*, 2009). The homologue of *Os*PTR2 was recently detected at the *q-28* locus (*Os*PTR9) for spikelet number per panicle (a key determinant of grain yield) in the same rice association panel as that used in this study (Rebolledo *et al.*, 2016). Likewise, Q4 was close (34 kb from peak SNP) to serine–threonine kinase (*Os*STE). The Arabidopsis orthologue of *Os*STE (*At*STE or BLUS1) is the major regulator of stomatal opening (Takemiya *et al.*, 2013; Supplementary Table S9).

### Seven grain-yield loci revealed a small to medium allelic effect in response to reproductive-stage water deficit

**Fig. 5.**
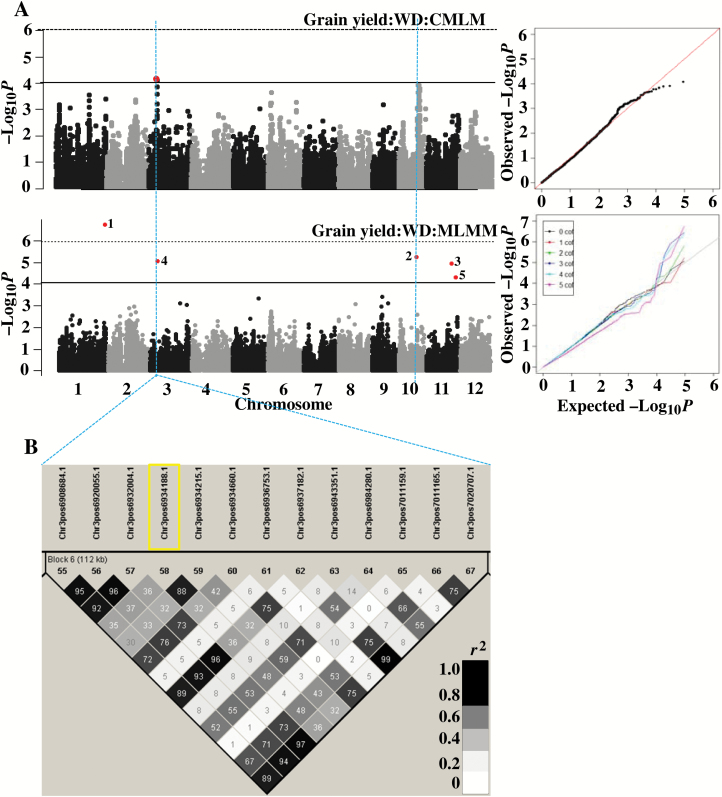
(A) GWAS results (Manhattan and quantile–quantile plot) detected through single-locus compressed mixed linear model (CMLM) and multi-locus mixed model (MLMM) for grain yield in 2014 water-deficit stress (WD) conditions. Significant SNPs on the Manhattan plot of MLMM are numbered according to the order in which they included as a cofactor in regression model. (B) Identified LD block (112 kb) based on *r*^2^ value between SNPs on chromosome 3 and the colour intensity of the box on the LD plot corresponds with *r*^2^ (multiplied by 100) according to the legend. Significant SNP marked by a rectangle was detected by CMLM and MLMM.

We identified two loci (Q9 and Q10) for grain yield under stress in 2013. The minor allele of both these loci had a negative effect on yield. Five significant loci Q11–Q15 were detected for yield under stress in 2014 (Fig. 5). The minor allele of Q11, Q12, and Q15 had a positive effect on yield, while the minor allele of two loci, Q13 and Q14, had a negative effect on yield. Q9 and Q10 harboured 18 and Q11–Q15 harboured 16 *a priori* candidate genes within the expected LD block region (Table 3). Seven *a priori* candidate genes, mostly near significant SNPs, are given in Supplementary Table S9. The Q9 locus was close (13 kb) to the phosphomannomutase gene regulating L-ascorbic acid biosynthesis and response to abiotic stress stimulus (Gene Ontology (GO):0009628). L-Ascorbic acid acts as a redox buffer to detoxify reactive oxygen species (ROS) (Arrigoni and De Tullio, 2002). Q11 was close to squalene monooxygenase or epoxidase (16 and 23 kb; two copies in LD block) and response to abiotic stress stimulus (GO:0009628). This gene is known to regulate ROS, stomatal responses and water-deficit tolerance in Arabidopsis (Posé *et al.*, 2009).

### Only three loci for grain yield acted via change in seed set percentage

**Fig. 6.**
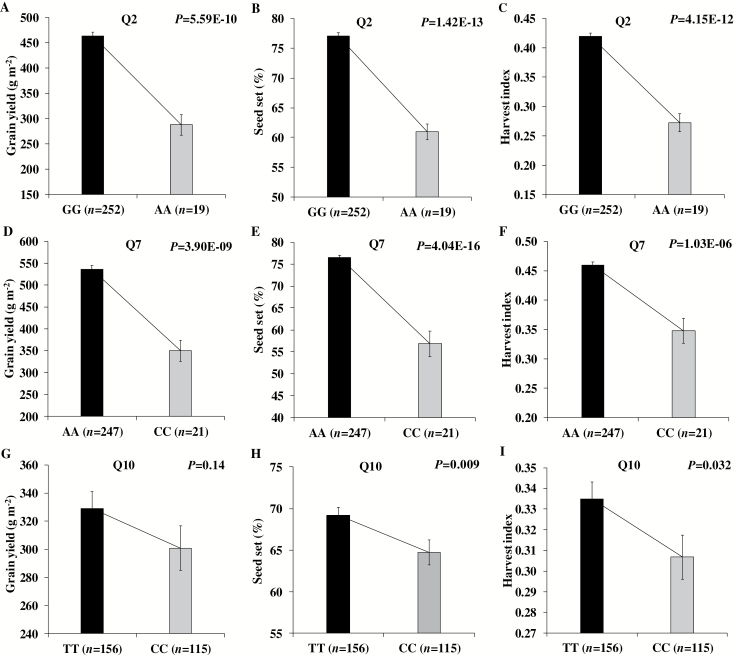
Allelic effect of Q2 (A–C; 2013), Q7 (D–F; 2014) in non-stress and Q10 (G–I; 2013) in water-deficit stress conditions on grain yield, seed set, and harvest index. Allelic effect of Q7 on harvest index was significant regardless of GWAS significance. Two-sample *t*-test *P*-value shows significant allelic effect difference with reference to major and minor allele. The Q10 locus also coincided with days to flowering.

**Fig. 7.**
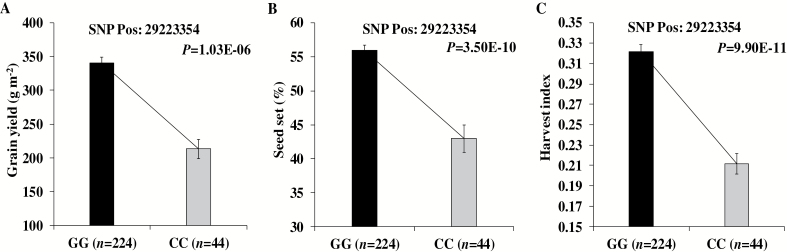
Allelic effect of chromosome 1 locus (29223354) on grain yield (A), seed set (B), and harvest index (C) in 2014 water-deficit stress conditions. Allelic effect on grain yield was significant regardless of GWAS significance. Two-sample *t*-test *P*-value shows significant allelic effect difference with reference to major and minor allele.

Although rice grain yield is co-determined by panicle number, spikelets per panicle, seed set percentage, and grain weight, very few loci of these component traits co-located with loci for yield *per se.* The seed set percentage is one of the most important yield components as indicated by its strong correlation with yield (Supplementary Figs S4, S5). Three loci were regulating yield through changes in seed set percentage, i.e. two loci designated as Q2 (2013) and Q7 (2014) in non-stress, and Q10 (2013) in stress conditions. The major allele (allele refers to the 0.95 frequency in the studied population) of these loci had a respective positive effect on yield, seed set, and harvest index (Fig. 6). In addition, the Q10 was also detected for days to flowering. No loci were common for yield and seed set in 2014 stress conditions, but one of the loci on chromosome 1 (29223354) was commonly detected for seed set and harvest index (Supplementary Fig. S7). Similarly, the major alleles had a respective positive effect on seed set, harvest index, and yield (irrespective of genetic significance) (Fig. 7). Hence, these loci were regulating yield through the effect of seed set on harvest index.

Four *a priori* candidate genes were predicted within the expected LD block of these loci. The Q2 was close (55 kb from peak SNP) to the plastocyanin gene that regulates flower development (GO:0009908) and pollination (GO:0009856) in rice (Supplementary Table S9). The Arabidopsis orthologue of this gene regulates seed set and pollen tube growth (Dong *et al.*, 2005). Q7 was within the novel expressed protein, which provides an entry point for future study. Sugar transport or uptake is essential for normal pollen development (Reinders, 2016), while the lack of starch synthesis arrests the pollen development in water deficit conditions thereby regulating seed set (Sheoran and Saini, 1996). Our Q10 locus was within the sugar transporter gene that plays an important role in sugar distribution. The rice grain yield *MQTL*_*2.1*_ (meta-analysis QTL) detected in water-deficit conditions also contained the sugar transporter gene (Swamy *et al.*, 2011). Similarly, the locus on chromosome 1 for seed set and harvest index in 2014 stress was near (34 kb from peak SNP) to the nitrate transporter gene that plays a role in rice yield increment (Fan *et al.*, 2016).

## Discussion

The main aim of this study was to link phenotypic variation with genetic markers, thereby gaining insights about promising candidate genes and the genetic architecture controlling yield traits. To the best of our knowledge, this is the first study conducted on the rice PRAY association mapping panel under reproductive-stage water-deficit stress. The key findings from our study are discussed below.

### Statistical trait adjustment can reduce confounding effect of desynchronized flowering on genetic analysis under water deficit

The desynchronized flowering time may result in the identification of QTLs, often colocating with QTLs for phenology and grain yield in reproductive-stage stress (Pinto *et al.*, 2010). Our genetic analysis of statistically corrected trait values was effective in minimizing the effect of desynchronized flowering time, as it led to detection of several novel loci that were not detected for non-corrected trait values. Despite statistical adjustment for flowering time, our novel Q10 for grain yield was co-localized with flowering time (different SNPs but falling within the same gene and LD block). In addition, it was also co-localized with seed set and harvest index. Previous studies in rice have identified several grain yield QTLs using linkage mapping under reproductive water-deficit stress conditions (Bernier *et al.*, 2007; Venuprasad *et al.*, 2009; Vikram *et al.*, 2011; Swamy *et al.*, 2013; Mishra *et al.*, 2013), of which some co-localized with plant height (*qDTY*_*6.2*_), days to flowering (*qDTY*_*3.2*_), or both (*qDTY*_*1.1*_). Interestingly, the major effect of *qDTY*_*1.1*_ was consistent even after statistical correction of grain yield using flowering time and plant height as covariates (Vikram *et al.*, 2011), and the recent detailed characterization confirmed the tight linkage and not the pleiotropy of this QTL with plant phenology (Vikram *et al.*, 2015). Our novel Q10 provided higher confidence of a causative SNP placed directly within the sugar transporter gene. However, this SNP was just 5 kb away from the COP9 signalosome complex subunit 4 gene within the same LD block (Supplementary Table S9). The COP9 signalosome complex gene is known to regulate flower development in Arabidopsis (Wang *et al.*, 2003), although the role of this gene in rice flowering has not been reported. Therefore, a further characterization of Q10 would be interesting to decipher the relationship with flowering time and stress tolerance to test linkage versus pleiotropy. Nevertheless, the effect of our consistent Q9 for grain yield (detected using either corrected or non-corrected values) was independent of flowering time stress conditions. More precise flowering time synchronization in 2014, which allowed identification of the genetic loci without having any co-localization with flowering time in stress conditions, added value to the findings. To the best of our knowledge, this is the first report demonstrating the effectiveness of better synchronization of flowering time phenology on a large GWAS panel under stress conditions at field level.

### Genetic control of grain yield, its components, and related traits was mostly independent and environment-specific

Grain yield is a complex trait determined by many interactive physiological processes changing temporally during the growing period. These processes often match the development of the key yield components in cereals that are genetically less complex than yield *per se* (Yin *et al.*, 2002). In rice, grain yield is the product of the panicle number or productive tiller (determined during the vegetative phase), spikelets per panicle (determined during panicle initiation), seed set percentage (determined during gametogenesis and anthesis), and individual grain weight (determined during grain filling). The genetic selection for each of these traits during rice domestication has given rise to rich genetic diversity (Doebley *et al.*, 2006; Sweeney and McCouch, 2007). To date, molecular genetic studies have detected QTLs underlying these genetic changes in rice yield components (http://www.gramene.org/). From these QTLs some of the candidate genes were successfully identified, notably displaying improvement in grain yield (Ashikari *et al.*, 2005; Fan *et al.*, 2006; Song *et al.*, 2007; Shomura *et al.*, 2008; Huang *et al.*, 2009; Miura *et al.*, 2010). For instance, the *SPIKE* gene/allele regulating the spikelet numbers indicated 13–36% yield increment in rice (Fujita *et al.*, 2013). In the present study, genetic dissection of these yield components enabled us to detect more loci than yield *per se* that were directly or indirectly contributing to rice grain yield. The co-localization of grain yield loci with yield components was limited in this study compared with other studies in rice (Lanceras *et al.*, 2004). This could be due to compensation among the yield components. In addition, these results emphasize the need for genetic analysis of yield components to identify additional genetic determinants having indirect effect on grain yield, providing alternative routes to enhance yield under water deficit.

Except for one locus on chromosome 12 for spikelets per m^2^ in 2014, the majority of the loci for grain yield and its component traits were specific to non-stress or stress conditions in both years. These results are in agreement with previous studies in rice (Lanceras *et al.*, 2004; Vikram *et al.*, 2011; Kumar *et al.*, 2014) and other crop species (Yin *et al.*, 2002; Millet *et al.*, 2016). Hence, the greater dependence on environments appeared to be a common characteristic of QTLs, although this does not negate their importance in marker-assisted selection. Despite strong variation in weather, we also detected four consistent loci: one each for panicles per m^2^ and spikelets per panicle on chromosomes 10 (19903199) and 4 (23423399), respectively, and two loci on chromosomes 2 (30699332) and 5 (5366489) for thousand-grain weight across years in non-stress conditions (Supplementary Table S7). These consistent regions with favourable alleles could be used for improving yield potential.

### Few overlaps of genetic loci with previously identified markers using same diversity panel

The PRAY population has been previously used in GWAS for a range of phenotypic traits (Qiu *et al.*, 2015; Al-Tamimi *et al.*, 2016; Rebolledo *et al.*, 2016; Kikuchi *et al.*, 2017; Kadam *et al.*, 2017). When comparing our results with these previous studies, we could not find any overlap between significant markers, except a SNP marker detected for plant height (position: 38286772) on chromosome 1, which was detected in our previous study (Kadam *et al.*, 2017). The most likely reasons for this lack of co-localization are difference in type and timing of stress or growing environments (QTL×environment interaction), population size, and molecular marker data used by previous studies or novel GWAS analysis methods (multi-locus) that are used in this study. Therefore, to make a more logical comparison for the same traits, we reanalysed the number of spikelets per panicle from Rebolledo *et al.* (2016) and yield and yield components from Kikuchi *et al.* (2017), using the same SNP datasets and analysis methods that are used in this study. This comparative analysis identified one locus on chromosome 2 (30518548) for panicle weight (equivalent to grain yield) from Kikuchi *et al.* (2017) that overlapped with grain yield locus (Q2: 30 523 925; different SNP but falls within the same LD block; Table 3; Supplementary Table S10) from 2013 non-stress conditions. In addition, there was also no overlap of a significant marker for grain yield and its components when comparing with other studies using different mapping panels under reproductive-stage water deficit (Ma *et al.*, 2016; Pantalião *et al.*, 2016; Swamy *et al.*, 2017). The major reasons for this were different rice genotypes or population size and inherent environmental and field variation for stress treatment (QTL×environment interaction). Another possible reason could be use of indica subspecies genotypes in this study while previous studies either used japonica subspecies (Pantalião *et al.*, 2016) or small population size (75 genotypes) with simple sequence repeat markers (Swamy *et al.*, 2017). In addition, it can be difficult to identify genomic regions or genes determining the trait difference across subspecies or genotypes.

### Seed set regulates the assimilate partitioning and grain yield

Better optimization of assimilate partitioning to reproductive organs with minimal competition among reproductive organs is essential to achieve stable and higher grain yield. So far, the physiological and genetic basis of the above processes have been poorly understood in rice and other cereal crops. Our study showed that the co-localization of grain yield loci with its components was rare. However, four genetic loci, namely Q2 and Q7 in non-stress, and Q10 and 29223354 (SNP position) in stress conditions, were regulating the grain yield and harvest index through changes in the seed set (Figs 6, 7). This indicates that the seed set is a critical determinant of assimilate partitioning (harvest index), thereby regulating the final expression of grain yield. A recent GWAS analysis confirmed these interactions in wheat (Guo *et al.*, 2017). Hence, these identified loci could be pyramided into an ‘ideotype’ at genomic level through marker-assisted selection to enhance rice grain yield in non-stress and stress conditions. In addition, such loci could also be of interest in identifying the physiological and molecular basis of assimilate partitioning to reproductive organs.

### Promising *a priori* candidate genes for grain yield and water-deficit stress resilience

We detected *a priori* candidate genes near peak SNP(s) within the LD block for grain yield loci (Supplementary Table S9). *A priori* candidate genes of grain yield loci can indicate possible roles of underlying physiological (SET kinase, sugar and nitrate transporter genes) and reproductive developmental (plastocyanin gene) processes in regulating the grain yield. Likewise, the abiotic stress tolerance candidate genes were detected near to grain yield loci in water-deficit conditions, of which genes regulating the detoxification of ROS (phosphomannomutase and squalene epoxidase genes) seem to be critical in rice stress tolerance (Selote and Chopra, 2004; Pyngrope *et al.*, 2013). These candidate genes need to be considered to detect the most likely causal genes. However, detailed large-scale molecular validations need to be conducted using the available approaches of RNAi, knockout mutants transgenic overexpression, and gene editing. Similarly, the loci for components and related traits that were not co-localized with yield *per se* could also be interesting candidates for further identification of novel genes.

## Concluding remarks

This study provides novel genetic loci for rice grain yield, its components, and related traits under non-stress and stress conditions in field phenotyping experiments. We detected several favourable alleles regulating these traits that, upon validation, can be effectively used in improving yield. Additional genetic loci with less overlap of yield component traits to yield *per se* clearly indicate the independent genetic regulation of these traits. Thus, many loci for component traits had an indirect effect on yield, which cannot be detected while mapping yield directly. This indicates the complexity of yield as a trait despite moderate to high heritability, which is often used as a selection criterion to improve yield potential and stress tolerance. Hence, future studies should also explore the genetic basis of individual component traits that are genetically less complex—an approach expected to give additional useful information to further enhance yield.

## Supplementary data

Supplementary data are available at *JXB* online.

Fig. S1. Field set-up of 296 genotypes screened under non-stress and reproductive-stage water-deficit stress in 2013 and 2014 experiments.

Fig. S2. Soil moisture tension measured using tensiometers in water-deficit stress field during 2013 and 2014, and rainfall pattern measured during stress period in 2013 and 2014.

Fig. S3. Climate parameters observed during the growing period.

Fig. S4. Pearson correlation coefficient between grain yield and its components and related traits in 2013 non-stress and water-deficit stress conditions.

Fig. S5. Pearson correlation coefficient between grain yield and its components and related traits in 2014 non-stress and water-deficit stress.

Fig. S6. GWAS results (Manhattan and quantile–quantile plot) detected through single-locus compressed mixed linear model and multi-locus mixed model for non-corrected and corrected harvest index (using days to flowering as a covariate) in 2013 water-deficit stress conditions.

Fig. S7. GWAS results (Manhattan and quantile–quantile plot) detected through single-locus compressed mixed linear model and multi-locus mixed model for seed-set and harvest index in 2014 water-deficit stress conditions.

Table S1. Summary statistics of grain yield and its components and related traits in 2013 and 2014 non-stress and water-deficit stress conditions.

Table S2. Multiple linear regression of grain yield with its components and related traits in non-stress and water-deficit stress conditions during 2013 and 2014.

Table S3. The details of genetic loci detected for grain yield components and related traits in 2013 non-stress conditions using compressed mixed linear-model and multi-locus mixed model methods.

Table S4. The details of genetic loci detected for uncorrected grain yield, its components, and related traits in 2013 water-deficit stress conditions using compressed mixed linear-model and multi-locus mixed model methods.

Table S5. The details of genetic loci detected for grain yield components and related traits in 2014 non-stress conditions using compressed mixed linear-model and multi-locus mixed model methods.

Table S6. The details of genetic loci detected for grain yield components and related traits in 2014 water-deficit stress conditions using compressed mixed linear-model and multi-locus mixed model methods.

Table S7. Common genetic loci detected across treatments (non-stress versus water-deficit stress) in 2013 or 2014 (A). Similarly, common genetic loci detected across years (2013 versus 2014) in NS or WD conditions (B).

Table S8. The details of genetic loci detected for corrected grain yield components and related traits (only on harvest index excluding the other traits in this group) in 2013 water-deficit stress conditions using compressed mixed linear-model and multi-locus mixed model methods.

Table S9. The list of *a priori* candidate genes within the linkage disequilibrium block of GWAS significant peak SNP/loci for grain yield in non-stress and water-deficit stress conditions.

Table S10. The details of genetic loci detected from previously published data on grain yield and yield components from Kikuchi *et al.* (2017), and number of spikelets per panicle (a key yield component) from Rebolledo *et al.* (2016), using the same rice PRAY panel.

Supplementary Figures and Tables
