# Supplementary material for: Genome-wide association reveals novel genomic loci controlling rice grain yield and its component traits under water-deficit stress during the reproductive stage
Source: J Exp Bot. 2018 May 15;69(16):4017–32. doi: 10.1093/jxb/ery186 (PMC6054195; doi:10.1093/jxb/ery186)
Supplement: Supplementary Figures and Tables [file ery186_suppl_supplementary_figures_and_tables.pdf]

Title: Genome wide association provides novel genomic loci controlling rice grain yield and its component traits under water-deficit stress during the reproductive stage

Supplementary Data

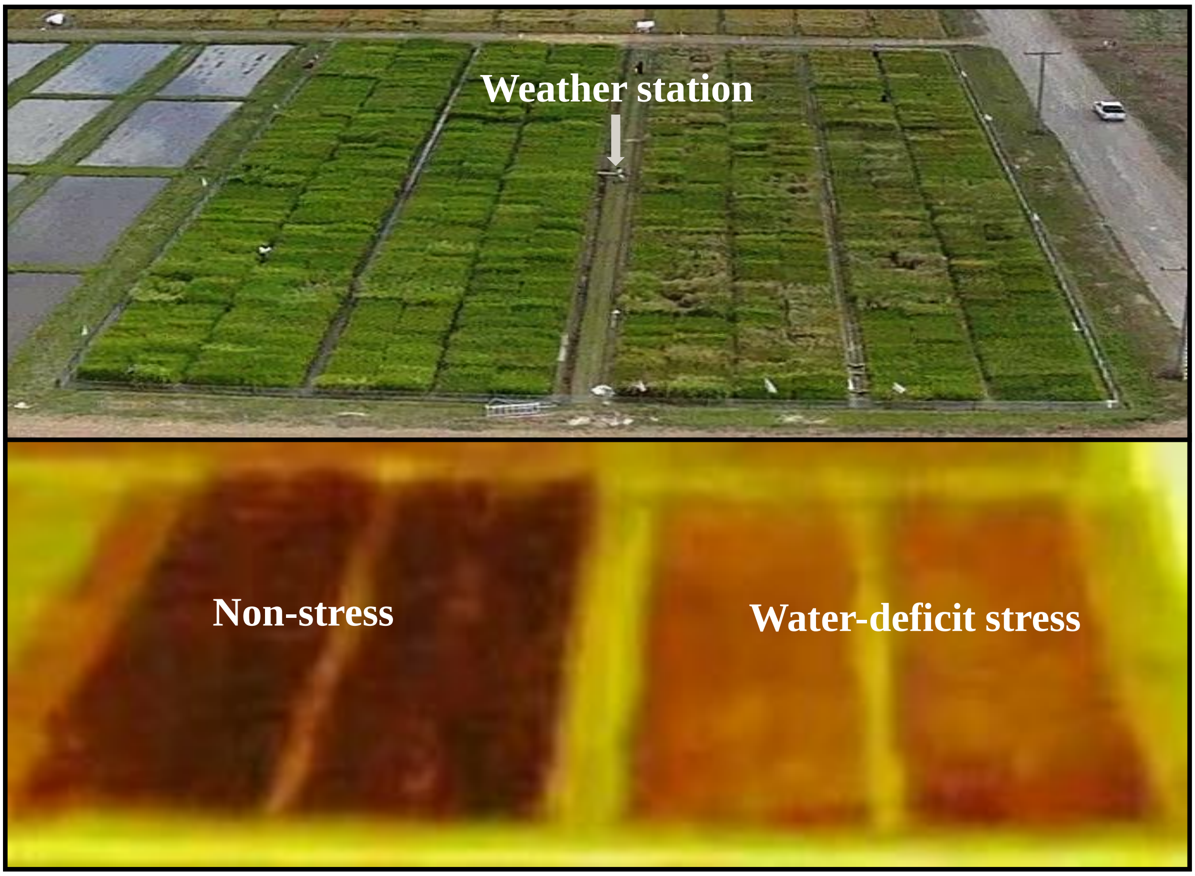

Supplementary Figure S1: Field set-up of 296 genotypes screened under non-stress and reproductive stage water-deficit stress in 2013 and 2014 experiments. Aerial picture of experiment plot taken in 2014 and lower panel was the thermal image taken during stress period showing canopy temperature difference in non-stress and water-deficit stress conditions.

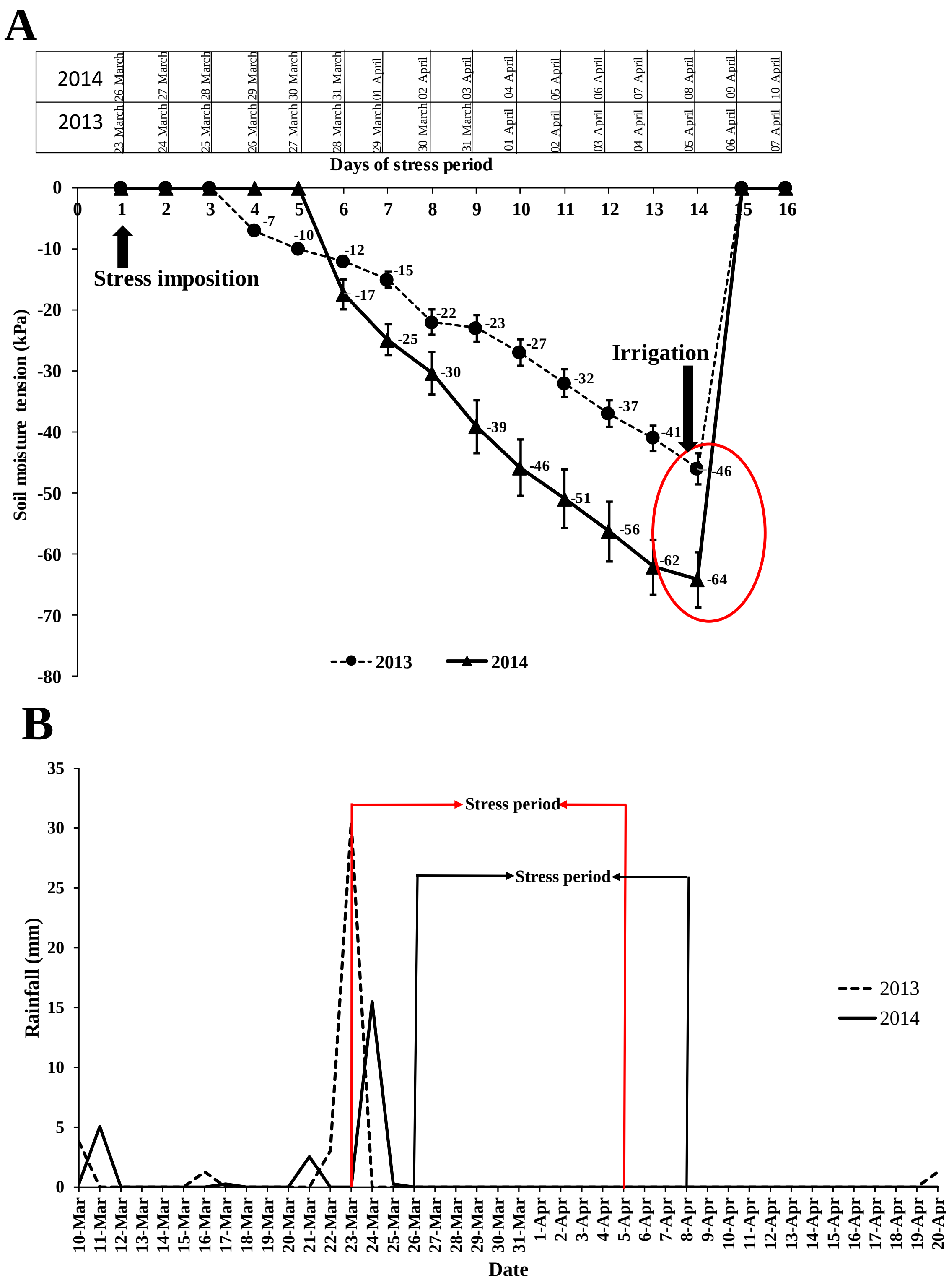

Supplementary Figure S2: Soil moisture tension measured using tensiometers in water-deficit stress field during 2013 and 2014 (**Panel A**), and rainfall pattern measured during stress period in 2013 and 2014 (**Panel B**). Soil moisture was measured using the 26 tensiometers randomly placed in the stress field at 30 cm depth and numbers above the symbols in Fig 2A are the average soil moisture tension from 26 tensiometers.

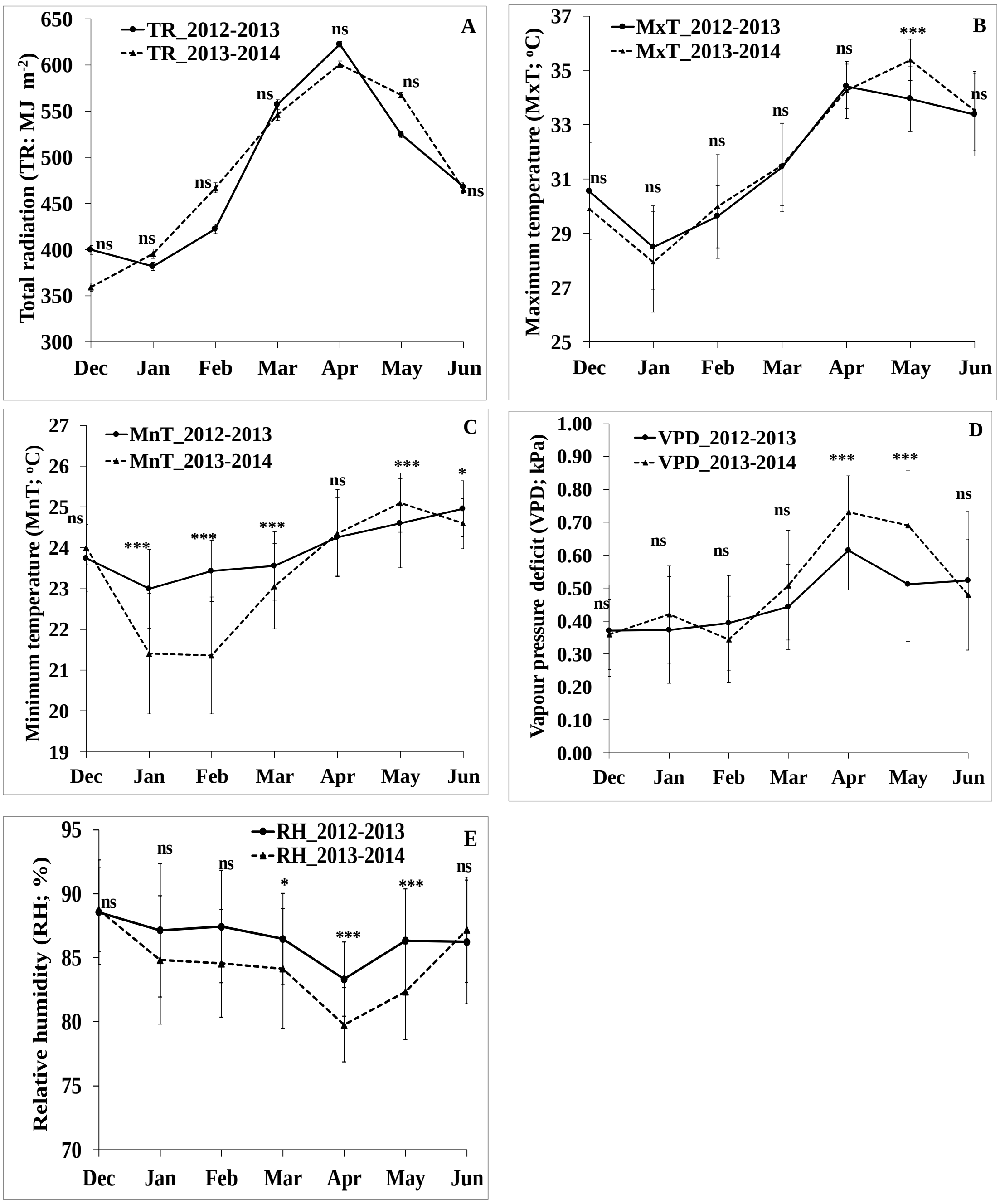

Supplementary Figure S3: Climate parameters observed during the growing period: Total radiation (**Panel A**), maximum temperature (**Panel B**), minimum temperature (**Panel C**), vapour pressure deficit (**Panel D**) and relative humidity (**Panel E**). Bar represent standard deviation. Paired t-test *P* value is used to compare the monthly climate difference across years with significance level of **P*<0.05, ***P*<0.01, ****P*<0.001, and ns = non-significant.

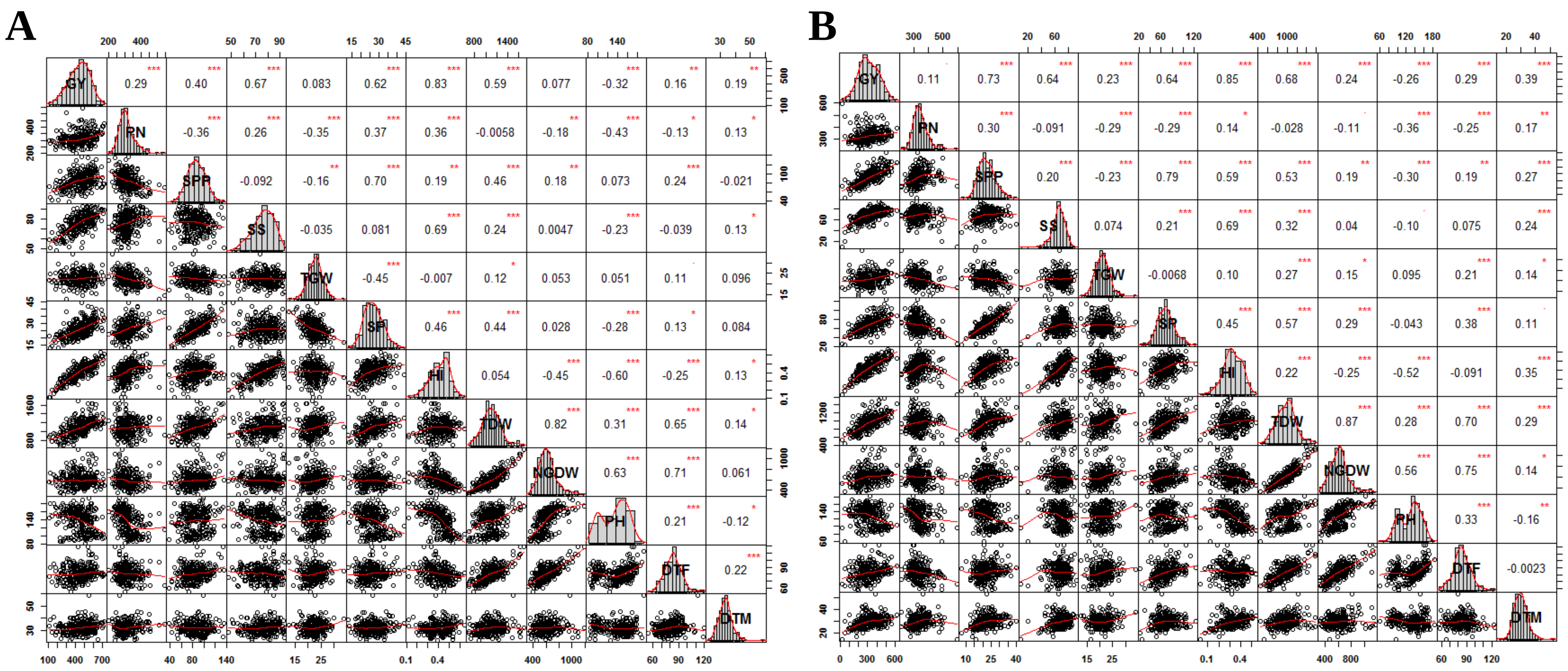

Supplementary Figure S4: Pearson correlation coefficient between grain yield and its components and related traits in 2013 non-stress **(Panel A)** and water-deficit stress **(Panel B)** conditions. Phenotypic traits with their histograms are given in the diagonal panel. Lower diagonal panel represents the scatter plot with red line depicting the best fit. The upper panel represents the Pearson correlation coefficient value and size of the correlation coefficient is proportional to the strength of the correlation. The correlation coefficient significance level: **P*<0.05, ***P*<0.01, ****P*<0.001. GY=grain yield; PN=panicles per m^2; SPP=spikelets per panicle; SS=seed set; TGW=thousand grain weight; SP=spikelets per m^2; HI=harvest index; TDW=total dry weight; NGDW=non-grain dry weight; PH=plant height; DTF= days to flowering; DTM=days to maturity.

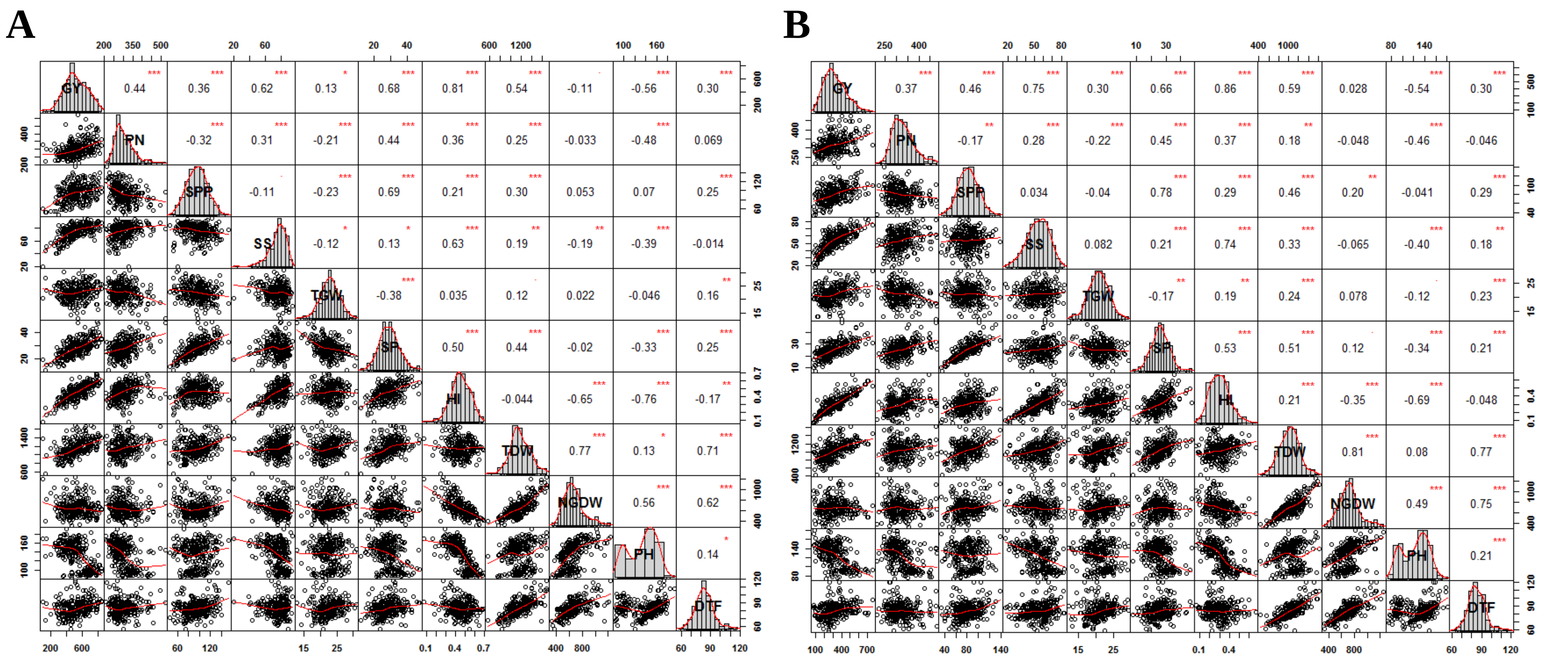

Supplementary Figure S5: Pearson correlation coefficient between grain yield and its components and related traits in 2014 non-stress **(Panel A)** and water-deficit stress **(Panel B)**. Phenotypic traits with their histograms are given in the diagonal panel. Lower diagonal panel represents the scatter plot with the red line depicting the best fit. The upper panel represents the Pearson correlation coefficient value and size of the correlation coefficient is proportional to the strength of the correlation. The correlation coefficient significance level: **P*<0.05, ***P*<0.01, ****P*<0.001. GY=grain yield; PN=panicles per m^2; SPP=spikelets per panicle; SS=seed set; TGW=thousand grain weight; SP=spikelets per m^2; HI=harvest index; TDW=total dry weight; NGDW=non-grain dry weight; PH=plant height; DTF= days to flowering.

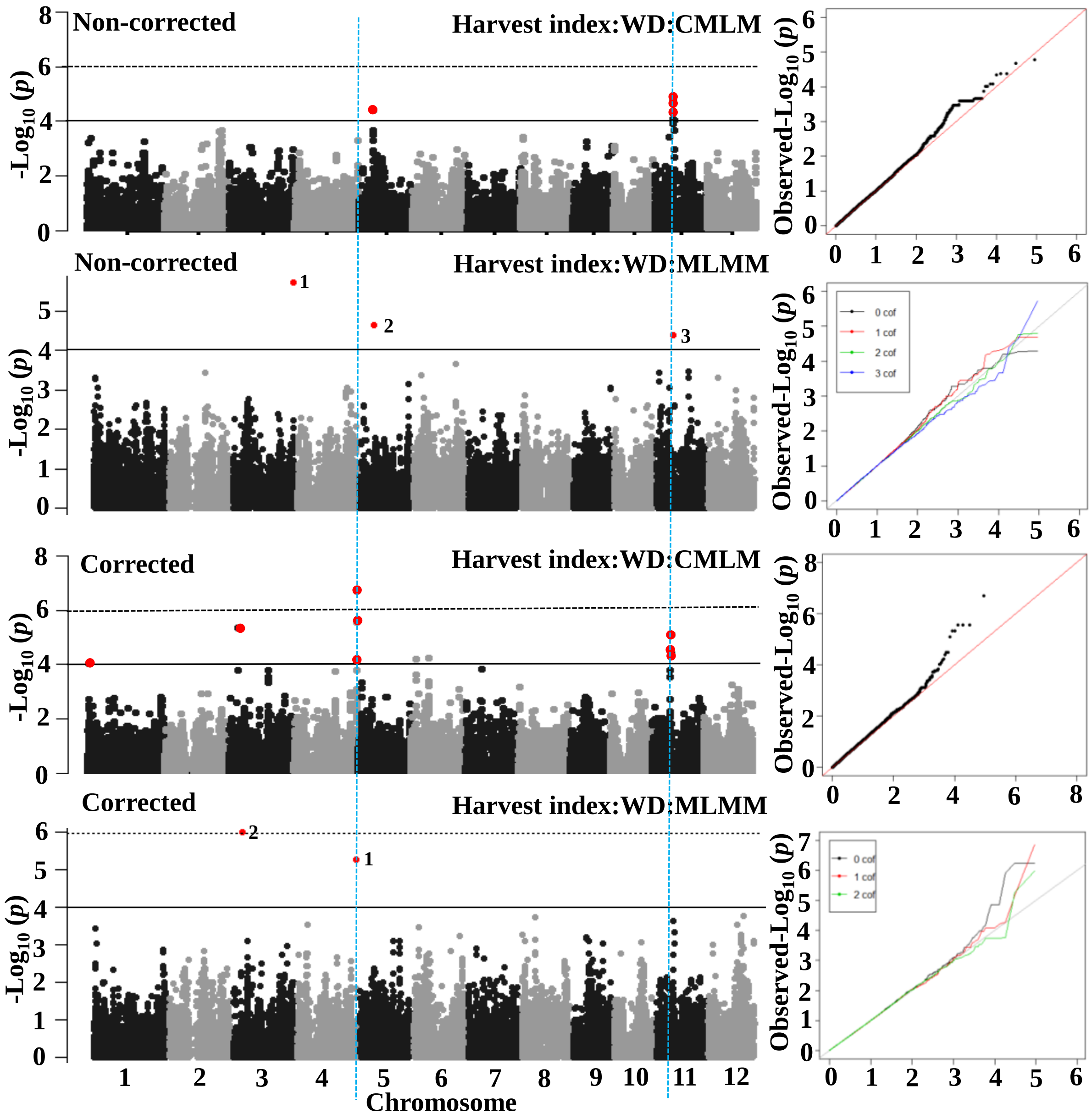

Supplementary Figure S6: GWAS results (Manhattan and Quantile-Quantile plot) detected through single-locus compressed mixed linear model (CMLM) and multi-locus mixed model (MLMM) for non-corrected and corrected harvest index (using days to flowering as a covariate) in 2013 water-deficit stress (WD) conditions. Significant SNPs in Manhattan plot of MLMM were numbered according to order in which they were included as a cofactor in regression model.

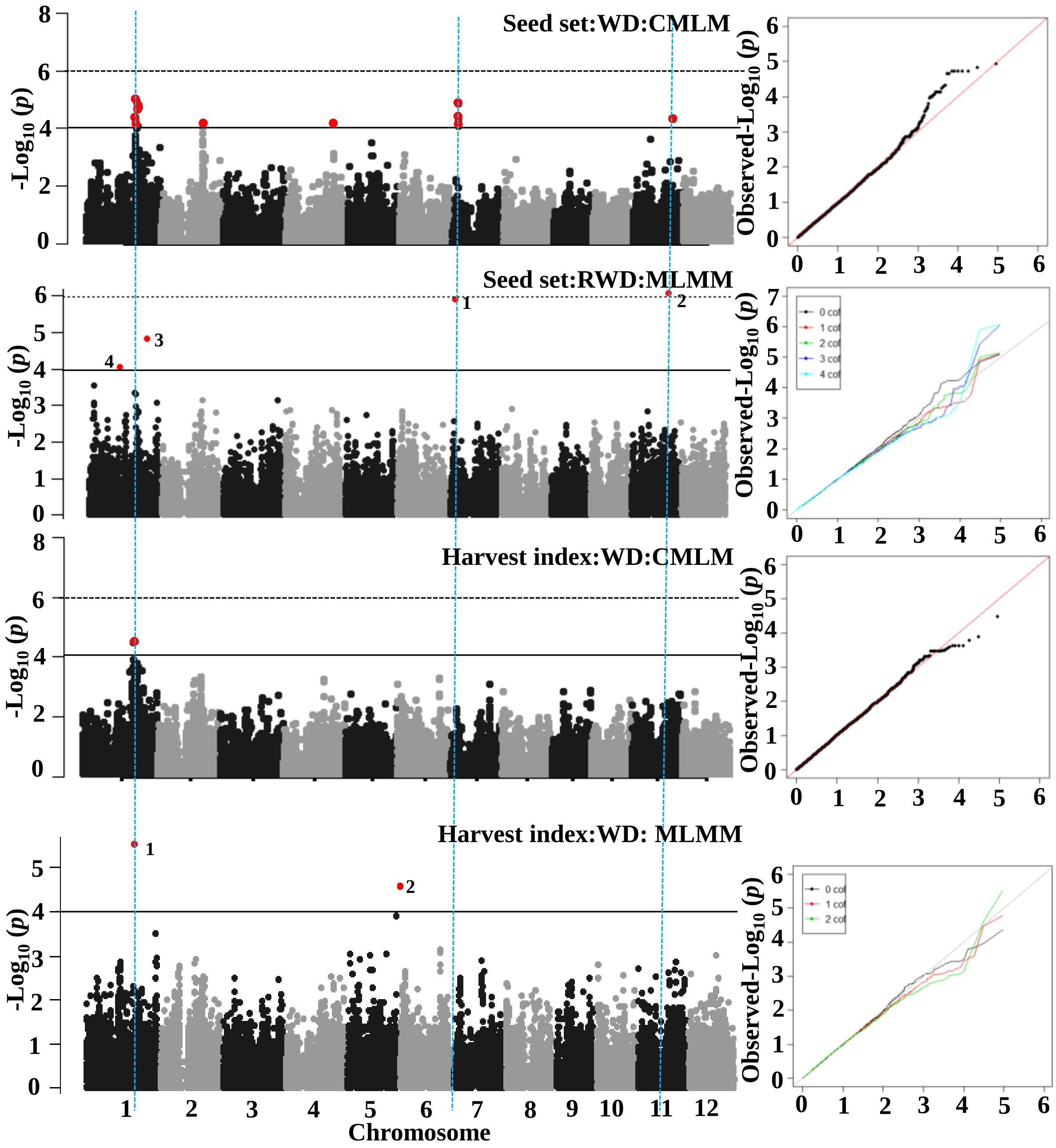

Supplementary Figure S7: GWAS results (Manhattan and Quantile-Quantile plot) detected through single-locus compressed mixed linear model (CMLM) and multi-locus mixed model (MLMM) for seed set and harvest index in 2014 water-deficit stress (WD) conditions. Significant SNPs in Manhattan plot of MLMM were numbered according to order in which they were included as a cofactor in regression model.

Supplementary Table S1: Summary statistics of grain yield and its components and related traits in 2013 and 2014 non-stress (NS) and water-deficit stress (WD) conditions.

		2013					2014				
**Trait**	**Trt**	**M±SD**	**Min**	**Max**	**H^2**	**h^2**	**M±SD**	**Min**	**Max**	**H^2**	**h^2**
GY	NS	451.1±123.2	106.3	727.0	0.92	0.27	521.9±143.3	102.8	839.7	0.93	0.75
	WD	317.3±112.9	16.7	622.6	0.84	0.64	319.5±134.3	78.1	761.1	0.73	0.73
**Grain yield component traits**											
PN	NS	316.8±56.7	200.8	540.8	0.82	0.88	302.7±51.7	212.0	520.3	0.89	0.92
	WD	340.4±69.5	219.2	593.6	0.87	0.87	321.0±50.0	215.1	479.2	0.80	0.90
SPP	NS	84.9±17.6	37.8	136.3	0.79	0.92	95.1±20.0	46.4	153.2	0.89	0.90
	WD	68.3±16.0	22.8	122.9	0.77	0.77	80.4±18.3	34.0	139.3	0.70	0.92
SS	NS	76.0±9.6	47.7	93.7	0.85	0.65	75.0±11.3	19.7	94.9	0.89	0.75
	WD	67.3±10.5	10.4	91.8	0.73	0.83	53.8±13.0	17.2	83.3	0.62	0.72
TGW	NS	22.5±2.9	12.5	34.0	0.99	0.86	22.6±3.0	12.9	30.9	0.99	0.88
	WD	20.9±3.0	12.6	34.1	0.97	0.85	21.1±3.4	12.3	29.1	0.77	0.90
SP	NS	26.3±5.4	13.1	43.8	0.88	0.88	28.4±6.2	11.7	47.8	0.91	0.92
	WD	22.5±5.3	7.3	40.1	0.85	0.84	25.5±6.3	7.6	47.7	0.83	0.93
**Other related traits**											
HI	NS	0.4±0.09	0.1	0.6	0.93	0.44	0.4±0.1	0.08	0.7	0.95	0.90
	WD	0.3±0.09	0.03	0.5	0.83	0.63	0.3±0.1	0.07	0.7	0.66	0.69
TDW	NS	1.1±0.1	0.7	1.7	0.87	0.80	1.1±0.2	0.5	1.7	0.89	0.80
	WD	0.9±0.2	0.4	1.5	0.90	0.87	1.0±0.2	0.4	1.6	0.86	0.83
NGDW	NS	0.6±0.1	0.3	1.1	0.93	0.94	0.6±0.1	0.3	1.2	0.92	0.90
	WD	0.6±0.1	0.3	1.1	0.92	0.90	0.7±0.1	0.3	1.2	0.89	0.89
PH	NS	136.6±27.6	81.8	193.9	0.98	0.88	135.2±27.8	84.3	191.4	0.98	0.86
	WD	128.1±25.6	59.7	184.1	0.97	0.90	125.0±27.0	74.0	280.1	0.90	0.86
DTF	NS	81.0±10.4	54.7	119.7	0.98	0.88	83.3±9.8	57.3	117.7	0.99	0.91
	WD	83.8±10.8	57.3	123.3	0.99	0.88	84.4±9.9	59.7	120.3	0.96	0.90
DTM	NS	32.3±4.7	21.7	59.0	0.84	0.31	-	-	-	-	-
	WD	29.7±4.7	14.8	53.0	0.86	0.47	-	-	-	-	-

Trt= treatment; M±SD= mean and standard deviation; Min= minimum; Max= maximum; H^2= broad-sense heritability; h^2= narrow-sense heritability. GY=grain yield; PN=panicles per m^2; SPP=spikelets per panicle; SS=seed set; TGW=thousand grain weight; SP=spikelets per m^2; HI=harvest index; TDW=total dry weight; NGDW=non-grain dry weight; PH=plant height; DTF= days to flowering; DTM=days to maturity. Data for days to maturity was not available for 2014 experiment.

Supplementary Table S2: Multiple linear regression of grain yield with its components and related traits in non-stress and water-deficit stress conditions during 2013 and 2014. Note that spikelets per m^2, harvest index and total dry weight were not included in the regression because spikelets per m^2 is the product of panicles and spikelets per panicles, and not an independent component. Similarly harvest index is the ratio of grain yield to total dry weight. Total dry weight is the sum of non-grain dry weight and grain dry weight.

**Year**	**Treatment**	**Traits**	***P*-value**	**R^2**
2013	Non-stress	Panicles	<0.001	0.89
		Spikelets per panicle	<0.001	
		Seed set	<0.001	
		Thousand grain weight	<0.001	
		Plant height	**0.069**	
		Days to flowering	**0.055**	
	Water-deficit	Panicles	<0.001	0.88
		Spikelets per panicle	<0.001	
		Seed set	<0.001	
		Thousand grain weight	<0.001	
		Plant height	<0.001	
		Days to flowering	<0.001	
2014	Non-stress	Panicles	<0.001	0.94
		Spikelets	<0.001	
		Seed set	<0.001	
		Thousand grain weight	<0.001	
		Plant height	<0.001	
		Days to flowering	<0.01	
	Water-deficit	Panicles	<0.001	0.93
		Spikelets per panicle	<0.001	
		Seed set	<0.001	
		Thousand grain weight	<0.001	
		Plant height	<0.001	
		Days to flowering	**0.404**	

Supplementary Table S3. The details of genetic loci detected for grain yield components and related traits in 2013 non-stress conditions using compressed mixed linear-model (CMLM) and multi-locus mixed model (MLMM) methods. The chromosome loci detected through both the methods were marked by asterisk sign (*); those detected through only by MLMM were marked by hashtag (#) sign. The remaining all unmarked chromosome loci were detected only through CMLM method.

Traits	Chr	Pos	Allele	MAF	*P* value$_{CMLM}$	*P* value$_{MLMM}$	AE	LD block		Size(kb)	Known genes
								Start	End		
**Grain yield component**											
PN	4	2481502	T:A	0.100	4.11E-05	-	28.53	2463707	2502427	38	2
	12	1691509	C:G	0.066	4.69E-05	-	27.81	1594015	1691770	97	12
	6	9369614	C:A	0.059	7.89E-05	-	32.90	9329842	9371867	42	4
	12	1691771	C:T	0.068	8.33E-05	-	26.60	1691771	1734838	43	7
	4	23514625	A:C	0.118	9.81E-05	-	24.72	23514625	23597658	83	3
	10#	19903199	T:A	0.148	-	8.47E-19	51.58	19882559	19916740	34	3
SPP	4*	23423399	A:G	0.288	4.70E-06	1.23E-07	-7.15	23423240	23512064	88	11
	11*	19641458	C:T	0.391	5.47E-05	1.20E-05	-5.13	19609894	19645174	35	4
	4	23417928	C:G	0.256	6.95E-05	-	-6.31	23357356	23417928	60	5
	3#	15094434	G:A	0.236	5.31E-06	-	9.21	14873722	15132484	258	17
	2#	24278919	G:C	0.332	2.13E-05	-	9.09	24265692	24283607	17	4
SS	2*	30523925	G:A	0.072	3.85E-05	5.56E-07	-7.55	30397910	30541202	143	16
	10#	18906753	G:C	0.303	-	1.76E-05	6.50	18898657	19018639	119	14
	2#	17591863	T:C	0.491	-	7.03E-05	-5.18	17591863	17806312	214	3
TGW	2	308723	A:T	0.458	5.35E-05	-	-0.73	221193	338309	117	8
	5*	5366489	G:A	0.387	5.45E-05	1.79E-05	0.82	5365520	5448285	82	6
	2*	30699332	T:C	0.295	7.75E-05	1.94E-05	0.81	30684655	30784063	99	9
SP	7*	22699138	T:C	0.185	3.18E-05	2.06E-05	2.04	22653977	22805994	152	12
**Other related traits**											
HI	8	20255596	G:T	0.063	5.45E-06	-	-0.06	20221039	20450490	229	12
	2*	30523925	G:A	0.072	7.68E-06	3.29E-10	-0.07	30397910	30541202	143	16
	8	20221030	G:A	0.068	1.12E-05	-	-0.05	20165675	20221035	55	5
	8	20160760	A:T	0.066	1.23E-05	-	-0.06	20144631	20165644	21	1
	10	2163454	C:T	0.493	7.00E-05	-	-0.02	2151405	2181552	30	1
	7#	17712506	T:C	0.24	2.79E-05	-	-0.05	17539335	17785193	245	7
TDW	4	21345052	G:C	0.063	1.84E-05	-	90.75	21337500	21360699	23	2
	5	26477176	A:G	0.063	9.24E-05	-	83.90	26473392	26847502	374	41
	4#	34815309	G:A	0.055	3.11E-05	-	-209.0	34815277	34833179	17	5
NGDW	2*	945729	A:T	0.225	1.14E-05	5.10E-07	43.23	944109	972602	28	4
	10	19874918	C:T	0.232	9.57E-05	-	36.54	19874875	19874918	44*bp*	0
	12#	24162384	G:C	0.306	2.18E-08	-	83.60	24070904	24389670	318	17
PH	1*	38286772	G:A	0.292	2.75E-07	9.46E-08	-12.37	38178239	38437530	259	29
	1	34203951	T:A	0.454	9.80E-05	-	5.99	34184887	34357192	172	6
DTF	3	21686259	T:C	0.185	5.26E-05	-	2.92	21660582	21686259	25	4
	3	21686358	T:C	0.185	5.26E-05	-	2.92	21686358	21944343	257	11
	3#	5113428	T:C	0.424	2.15E-05	-	-2.61	5021158	5167439	146	13
DTM	2*	19163866	T:G	0.093	5.91E-05	2.92E-05	2.08	19151240	19163870	12	1

Chr=chromosome; Pos= physical position of SNP; MAF=minor allele frequency; AE=allelic effect with reference to the minor allele (average traits value of genotypes carrying minor allele - average traits value of genotypes carrying major allele). LD= linkage disequilibrium. Known genes= total known genes observed within the LD block; PN=panicles per m^2; SPP=spikelets per panicle; SS=seed set; TGW=thousand grain weight; SP=spikelets per m^2; HI=harvest index; TDW: total dry weight.

Supplementary Table S4. The details of genetic loci detected for uncorrected grain yield, its components and related traits in 2013 water-deficit stress conditions using compressed mixed linear-model (CMLM) and multi-locus mixed model (MLMM) methods. The chromosome loci detected through both the methods were marked by asterisk sign (*); those detected through only by MLMM were marked by hashtag (#) sign. The remaining all unmarked chromosome loci were detected only through CMLM method. Trait acronyms are given in Table 1.

Traits	Chr	Pos	Allele	MAF	*P* value$_{CMLM}$	*P* value$_{MLMM}$	AE	LD block		Size (kb)	known genes
								Start	End		
GY	4*	34815277	C:T	0.074	1.17E-05	1.77E-06	-51.28	34815277	34833179	17	5
	9#	17886901	G:C	0.063	-	2.55E-06	89.60	17886901	18067376	180	15
	8#	1541432	C:T	0.133	-	3.88E-06	-107.75	1541432	1581667	40	5
	3#	8548868	A:G	0.439	-	4.06E-05	16.61	8532555	8673763	141	14
**Grain yield component traits**											
PN	12*	19257052	G:A	0.052	1.28E-06	1.84E-24	42.98	19188352	19339344	150	3
	4	31801144	C:G	0.066	3.57E-06	-	34.70	31784370	31819723	35	6
	6	27932410	A:G	0.055	3.87E-06	-	42.40	27930905	27935874	4	0
	6	27946105	T:C	0.057	1.48E-05	-	42.73	27939637	27954294	14	0
	2	29554958	G:A	0.114	1.90E-05	-	27.96	29550199	29554958	4	1
	6	29086891	C:T	0.055	2.71E-05	-	35.05	28969471	29138444	169	15
	6	880574	C:T	0.054	5.33E-05	-	38.32	818175	887142	68	9
	12	1691770	A:G	0.092	5.35E-05	-	27.49	1594015	1691770	97	12
	6	27918615	G:C	0.061	7.59E-05	-	38.18	27888568	27924404	35	1
	8	8333808	G:A	0.148	7.94E-05	-	24.50	8259055	8390193	131	7
	2	4668201	A:G	0.472	9.55E-05	-	-16.76	4668201	4711157	47	6
	12#	19121161	A:G	0.052	-	5.19E-18	8.52	19115304	19156989	41	5
SPP	7	22858259	C:T	0.203	6.74E-06	-	5.53	22815780	22877074	61	7
	7	22699138	T:C	0.185	1.35E-05	-	5.59	22653977	22805994	152	12
	4*	23423399	A:G	0.288	1.48E-05	1.66E-06	-5.68	23423240	23512064	88	11
	4	20084244	T:G	0.369	2.24E-05	-	4.17	20042539	20145355	102	8
	7	19654477	T:G	0.055	2.28E-05	-	8.01	19615905	19654863	38	2
	7	22815780	A:G	0.159	3.52E-05	-	5.50	22805994	22827392	21	2
	7	23086735	C:A	0.170	3.94E-05	-	5.11	22927040	23237152	310	26
	1	39831573	C:T	0.135	6.33E-05	-	5.82	39781249	39868654	87	13
	7*	21708194	T:C	0.125	8.61E-05	3.65E-05	5.30	21673763	21761328	87	9
SS	11	10232787	C:T	0.074	2.34E-05	-	-5.61	10204901	10235132	30	2
	11*	10131031	A:G	0.052	3.51E-05	4.64E-06	-6.15	9838715	10131062	292	3
	12*	27244607	G:A	0.055	4.04E-05	3.55E-08	-5.72	27093600	27244607	151	14
	11	10329677	C:A	0.055	5.65E-05	-	-6.24	10265286	10341103	75	2
	2	31546589	C:G	0.081	6.34E-05	-	-5.03	31397497	31602859	205	23
	11#	3111523	G:C	0.328	-	7.33E-06	1.50	2942864	3111772	168	7
	3#	35539634	C:T	0.151	-	1.90E-05	-3.00	35509880	35593310	83	8
TGW	2*	10359249	G:C	0.129	4.59E-05	2.03E-05	1.09	10205033	10369901	164	8
	3#	16725803	G:A	0.439	-	5.12E-05	1.57	16665467	16804385	138	6
	5#	5366489	G:A	0.387	-	6.06E-07	1.07	5366489	5448285	82	6
	1#	23384536	G:A	0.063	-	3.74E-05	-2.63	23299536	23430945	131	15
SP	12*	141599	G:A	0.122	3.07E-05	2.42E-05	-1.91	141599	148272	6	1
	7*	22827392	T:C	0.179	4.55E-05	1.29E-05	1.86	22815780	22877074	61	7
	7	21708194	T:C	0.125	6.64E-05	-	1.80	21673763	21761328	87	9
	7	22699138	T:C	0.185	8.87E-05	-	1.79	22653977	22805994	152	12
**Other related traits**											
HI	11	10232787	C:T	0.074	1.65E-05	-	-0.04	10204901	10235132	30	2
	5*	7978268	C:T	0.125	4.23E-05	2.32E-05	-0.04	7951244	8096795	145	8
	11	10131062	T:C	0.055	8.42E-05	-	-0.05	9838715	10131062	292	3
	11	10329677	C:A	0.055	8.42E-05	-	-0.05	10265286	10341103	75	2
	11	10627944	A:C	0.074	9.75E-05	-	-0.04	10627944	10863355	235	9
	11*	10392338	C:T	0.074	9.96E-05	4.00E-05	-0.04	10353380	10416332	62	1

	3[#]	35539634	C:T	0.151	-	1.93E-06	-0.03	35509880	35593310	83	8
TDW	4[*]	34815277	C:T	0.074	5.84E-05	2.15E-06	-89.16	34815277	34833179	17	5
	11[#]	16582568	C:T	0.203	-	5.54E-05	34.25	16511087	16623466	112	1
NGDW	1[*]	18626303	C:G	0.343	9.85E-05	6.28E-05	39.34	18626303	18888393	262	5
	2[#]	1006437	T:C	0.406	-	1.66E-06	58.94	1006427	1103758	97	15
	12[#]	24162384	G:C	0.306	-	3.61E-05	97.16	24070904	24389670	318	17
PH	1[*]	38286772	G:A	0.292	1.24E-07	3.15E-10	-11.76	38178239	38437530	259	29
	1	35548077	C:T	0.197	1.37E-05	-	-8.47	35504716	35595543	90	9
	1	35062897	C:T	0.205	5.86E-05	-	-7.57	35031550	35099986	68	6
	5	1910382	C:T	0.177	9.27E-05	-	5.62	1864314	2107292	242	22
	7[#]	58252	T:A	0.125	-	2.35E-06	-7.16	19107	134004	114	10
	4[#]	5806676	C:T	0.210	-	4.71E-05	3.99	5683343	5816801	133	7
DTF	12	1881367	T:A	0.109	5.58E-06	-	-4.63	1753092	1886677	133	18
	3	21670338	T:C	0.280	6.75E-06	-	3.55	21660582	21686259	25	4
	3	21944343	A:G	0.277	1.59E-05	-	3.39	21686358	21944343	257	11
	3	22056925	A:G	0.196	4.83E-05	-	2.99	22056925	22107644	50	2
	3	22164972	A:G	0.255	9.27E-05	-	3.15	22120547	22205824	85	4
	12	1691771	C:T	0.068	7.64E-06	-	-4.90	1691771	1734838	43	7
	12	1691509	C:G	0.066	7.80E-06	-	-4.86	1594015	1691770	97	12
	3[*]	5113535	G:C	0.310	2.05E-05	9.53E-06	-3.23	5021158	5167439	146	13
	11	23178024	C:T	0.085	2.77E-05	-	4.27	23178024	23183705	5	1
	3	21659472	A:G	0.251	2.99E-05	-	3.40	21315611	21660079	344	20
	1	15153868	C:A	0.175	9.03E-05	-	-3.30	15027451	15169454	142	7

Chr=chromosome; Pos= physical position of SNP; MAF=minor allele frequency; AE=allelic effect with reference to the minor allele (average traits value of genotypes carrying minor allele - average traits value of genotypes carrying major allele), LD= linkage disequilibrium. Known genes= total known genes observed within the LD block. GY=grain yield; PN=panicles per m^2; SPP=spikelets per panicle; SS=seed set; TGW=thousand grain weight; SP= spikelets per m^2; HI=harvest index; TDW= total dry weight; NGDW=non-grain dry weight; PH=plant height; DTF=days to flowering.

Supplementary Table S5. The details of genetic loci detected for grain yield components and related traits in 2014 non-stress conditions using compressed mixed linear-model (CMLM) and multi-locus mixed model (MLMM) methods. The chromosome loci detected through both the methods were marked by asterisk sign (*); those detected through only by MLMM were marked by hashtag (#) sign. The remaining all unmarked chromosome loci were detected only through CMLM method. Trait acronyms are given in Table 1.

Traits	Chr	Pos	Allele	MAF	*P* value$_{CMLM}$	*P* value$_{MLMM}$	AE	LD block		Size (kb)	Known genes
								Start	End		
**Grain yield component traits**											
PN	10	19650831	A:C	0.149	2.05E-06	-	19.35	19474964	19665687	190	14
	10*	19903199	T:A	0.146	2.41E-06	1.16E-06	19.55	19882559	19916740	34	3
	10	19713719	T:G	0.157	6.46E-06	-	17.98	19665880	19778630	112	11
	10	19463253	T:C	0.153	2.08E-05	-	16.79	19280939	19474522	193	21
	10	19107872	A:T	0.175	2.32E-05	-	17.78	19049329	19219240	169	21
	10	19788019	A:G	0.157	4.75E-05	-	16.93	19787326	19827002	39	1
	4*	28829512	G:A	0.101	6.10E-05	8.74E-05	21.71	28701604	29126558	424	31
	8	27367304	C:T	0.175	7.25E-05	-	15.97	27364583	27381695	17	5
	3#	30407838	C:G	0.295	-	1.18E-06	14.84	30407838	30499464	91	10
SPP	4*	23423399	A:G	0.287	2.44E-05	2.41E-05	-7.40	23423240	23512064	88	11
	11*	22233662	G:T	0.104	8.24E-05	5.36E-05	-8.48	22203908	22242728	38	2
SS	6*	1174802	T:C	0.084	8.61E-05	2.64E-08	-4.54	1117344	1208291	90	2
	10	22428992	A:T	0.052	8.77E-05	-	-5.57	22419446	22436125	16	3
	1#	19327482	T:C	0.183	-	1.09E-06	-14.32	19247447	19360189	112	6
	8#	10059172	G:A	0.056	-	1.72E-06	-4.12	9997926	10079318	81	4
	11#	19108095	A:G	0.224	-	1.77E-06	6.09	19075632	19130323	54	4
	12#	5105627	A:C	0.078	-	1.42E-05	-19.68	5101105	5390949	289	12
	2#	4862726	G:A	0.239	-	1.60E-05	-7.84	4774730	4869814	95	15
	4#	21381516	C:A	0.063	-	7.34E-05	2.37	21360906	21381516	20	5
TGW	2	30699332	T:C	0.291	4.21E-05	-	0.90	30684655	30784063	99	9
	2#	10359249	G:C	0.131	-	8.66E-08	2.42	10205033	10369901	164	8
	7#	22413176	C:G	0.396	-	3.01E-14	-1.04	22404388	22606330	201	14
	5#	5366489	G:A	0.388	-	1.32E-06	1.19	5365520	5448285	82	6
	3#	16736753	C:T	0.455	-	4.58E-06	-1.47	16665467	16804385	138	6
	3#	12760491	A:C	0.075	-	1.39E-05	2.38	12717890	12764642	46	5
SP	11*	18102156	A:C	0.295	6.13E-05	1.81E-06	-1.75	18095189	18129687	34	2
	12#	10443628	C:A	0.478	-	1.38E-06	2.26	10320934	10622432	301	10
	4#	4550145	C:T	0.313	-	1.41E-05	-0.47	4521317	4558051	36	1
	11#	22065446	T:C	0.134	-	2.08E-05	-5.65	22065446	22150769	85	3
**Other related traits**											
HI	10	22419446	A:G	0.056	1.71E-05	-	-0.05	22419446	22436125	16	3
	8	16617975	T:C	0.259	7.73E-05	-	-0.02	16611066	16619402	8	3
	1*	42643328	G:A	0.272	9.78E-05	3.36E-06	-0.02	42627969	42643337	15	3
	8#	16324317	A:T	0.097	-	2.04E-06	-0.10	16308107	16398979	90	5
TDW	2*	945729	A:T	0.224	4.92E-05	1.29E-06	63.09	944109	972602	28	4
	4#	30764890	A:G	0.354	-	2.59E-08	-3.62	30690751	30790417	99	7
	1#	143282	G:A	0.116	-	2.40E-05	20.85	19837	197790	177	16
	3#	33546549	C:A	0.291	-	4.08E-05	-40.9	33518876	33569432	50	5
NGDW	5*	21385305	C:T	0.09	1.37E-05	9.65E-05	76.41	21312370	21385305	72	4
	1*	42363099	C:T	0.101	1.37E-05	2.92E-05	73.35	42326848	42367533	40	3
	11	10867613	C:T	0.153	6.24E-05	-	58.86	10834263	10928827	94	3
	3	34032565	A:G	0.246	6.71E-05	-	-48.68	34004938	34069043	64	5
	7#	21850303	C:T	0.437	-	2.59E-05	100.45	21814261	21908474	94	13
PH	1*	38286772	G:A	0.291	6.03E-09	2.57E-14	-13.34	38178239	38437530	259	29
	1	34280616	G:A	0.455	9.54E-06	-	6.22	34184887	34357192	172	6
	1	35548077	C:T	0.196	2.51E-05	-	-8.04	35504716	35595543	90	9
	1	35062897	C:T	0.203	2.93E-05	-	-7.8	35031550	35099986	68	6

	1	33059505	T:A	0.332	4.31E-05	-	-5.36	32994632	33284067	289	30
	9$^{\#}$	20537268	C:T	0.078	-	3.52E-06	4.87	20428722	20537316	108	6
DTF	12	19403471	T:C	0.356	8.37E-05	-	-2.24	19400490	19412883	12	0
	3$^{\#}$	5113428	T:C	0.425	-	5.53E-09	-2.03	5021158	5167439	146	13
	3$^{\#}$	72105	C:T	0.086	-	1.36E-10	7.98	6480	197654	191	22
	3$^{\#}$	28533036	C:A	0.056	-	2.57E-09	13.33	28529762	28761862	232	28
	7$^{\#}$	21266079	C:T	0.090	-	2.12E-05	-4.68	21245869	21290877	45	3
	4$^{\#}$	30764890	A:G	0.354	-	1.01E-07	-0.05	30690751	30790417	99	7
	9$^{\#}$	11299373	C:T	0.164	-	8.11E-07	4.08	11299269	11315063	15	1
	12$^{\#}$	24162384	G:C	0.310	-	1.44E-07	6.04	24070904	24389670	318	17
	11$^{\#}$	18168801	G:T	0.052	-	8.16E-06	-7.33	18134653	18242389	107	1
	6$^{\#}$	10389819	G:T	0.071	-	2.17E-05	-4.58	10279684	10410579	130	8

Chr=chromosome; Pos= physical position of SNP; MAF=minor allele frequency; AE=allelic effect with reference to the minor allele (average traits value of genotypes carrying minor allele - average traits value of genotypes carrying major allele), LD= linkage disequilibrium. Known genes=total known genes observed within the LD block. PN=panicles per m^2; SPP=spikelets per panicle; SS=seed set; TGW=thousand grain weight; SP= spikelets per m^2; HI=harvest index; TDW= total dry weight; NGDW=non-grain dry weight; PH=plant height; DTF=days to flowering.

Supplementary Table S6. The details of genetic loci detected for grain yield components and related traits in 2014 water-deficit stress conditions using compressed mixed linear-model (CMLM) and multi-locus mixed model (MLMM) methods. The chromosome loci detected through both the methods were marked by asterisk sign (*); those detected through only by MLMM were marked by hashtag (#) sign. The remaining all unmarked chromosome loci were detected only through CMLM method. Trait acronyms are given in Table 1.

Traits	Chr	Pos	Allele	MAF	*P* value$_{CMLM}$	*P* value$_{MLMM}$	AE	LD block		Size (kb)	Known genes
								Start	End		
**Grain yield component traits**											
PN	11*	2170439	C:A	0.073	2.67E-05	3.21E-06	29.60	1940541	2291962	351	39
	1#	39886933	G:C	0.254	-	3.60E-05	-37.99	39886933	40061573	174	15
	3#	32507536	T:G	0.489	-	4.56E-05	-14.37	32507536	32594052	86	9
SS	1	29223354	G:C	0.164	1.17E-05	-	-5.16	29135405	29300574	165	13
	7*	3293128	G:T	0.172	1.46E-05	1.27E-06	5.18	3293128	3297350	4	2
	1	30657333	G:A	0.198	1.86E-05	-	-4.63	30583128	30819501	236	25
	1	29483935	T:A	0.104	2.22E-05	-	-5.94	29394012	29600705	206	19
	11*	23110189	G:A	0.063	4.73E-05	8.69E-07	-6.95	23066834	23133274	66	6
	1	28427790	C:T	0.097	5.69E-05	-	-5.47	28406849	28444503	37	1
	4	27444465	G:T	0.175	7.38E-05	-	-4.90	27316695	27568586	251	27
	2	23767444	G:T	0.067	7.47E-05	-	-6.78	23754037	23767444	13	3
	2	24234634	T:G	0.093	9.39E-05	-	-6.55	24228897	24236361	7	1
	1#	35548077	C:T	0.194	-	1.47E-05	11.27	35504716	35595543	90	9
	1#	19262986	G:A	0.138	-	8.75E-05	-2.72	19248831	19360189	111	6
TGW	1*	3398710	A:G	0.338	1.76E-05	9.80E-08	-1.14	3398710	3538828	140	16
	3	16725803	G:A	0.435	2.23E-05	-	1.23	16665467	16804385	138	6
	4	16574303	C:T	0.104	7.98E-05	-	1.58	16574289	16642369	68	5
	5#	7021512	C:T	0.063	-	2.94E-07	3.00	7021378	7039434	18	1
	1#	21362367	G:A	0.086	-	1.77E-08	-1.24	21348660	21627407	278	8
	3#	22959891	C:T	0.075	-	2.62E-06	2.16	22817514	22959891	142	7
	9#	17852034	A:G	0.06	-	1.10E-05	2.85	17844173	17979314	135	13
	3#	7043553	T:C	0.440	-	1.98E-05	-0.93	7030587	7070877	40	6
SP	12*	10611754	C:A	0.496	4.26E-05	1.01E-08	1.83	10320934	10622432	301	10
	12#	16565406	G:A	0.407	-	7.30E-05	2.77	16565406	16584455	19	1
	11#	3518037	G:A	0.101	-	6.62E-05	2.61	3368572	3562283	198	18
**Other related traits**											
HI	1*	29223354	G:C	0.164	3.31E-05	3.03E-06	-0.03	29135405	29300574	165	13
	6#	217858	C:T	0.119	-	2.59E-05	-0.001	132127	366436	234	25
TDW	3*	15532341	T:C	0.481	5.81E-05	3.76E-05	58.79	15532341	15564883	32	2
	12	23011365	A:C	0.067	7.82E-05	-	-102.57	23004415	23141150	136	13
	12	2589690	C:T	0.146	9.78E-05	-	-67.86	2567973	2594603	26	4
	6*	9774102	C:T	0.144	9.93E-05	1.01E-09	-75.66	9655595	9774102	118	2
	7#	27620959	C:T	0.090	-	4.12E-10	71.75	27479689	27620959	141	8
	1#	42643699	C:T	0.104	-	4.22E-06	-88.94	42627969	42691537	63	5
	7#	26457561	G:A	0.06	-	5.90E-06	248.12	26450722	26548855	98	12
	9#	6323526	G:A	0.299	-	6.60E-07	136.50	6195580	6323526	127	7
	10#	17454693	A:G	0.078	-	2.00E-06	134.78	17378773	17548721	169	9
	6#	2721526	A:T	0.231	-	1.94E-06	-39.32	2662180	2726347	64	10
	6#	7135140	A:G	0.306	-	1.90E-05	12.67	7110053	7136325	26	4
NGDW	11*	10867613	C:T	0.153	4.42E-05	2.44E-05	61.13	10834263	10928827	94	3
	7	3488686	C:G	0.312	5.84E-05	-	-55.51	3454851	3619076	164	15
PH	1*	38286772	G:A	0.291	1.39E-07	3.49E-08	-13.23	38178239	38437530	259	29
	2	23720396	C:G	0.295	4.62E-05	-	5.87	23720396	23720592	197*bp*	1
	11	586603	C:T	0.093	9.70E-05	-	-10.55	566590	642837	76	16
	5	16676691	C:T	0.070	2.86E-05	-	8.89	16588982	16785352	196	9
DTF	3*	72105	C:T	0.086	6.61E-08	3.74E-08	1.82	6480	197654	191	22

4[#]	23430194	T:C	0.257	-	6.09E-07	-3.18	23424327	23483270	58	6
1[#]	855970	G:C	0.172	-	8.52E-07	1.94	769982	931087	161	19
4[#]	34314696	G:T	0.052	-	2.23E-05	-2.33	34284403	34314696	30	3
12[#]	24162384	G:C	0.310	-	8.53E-06	5.83	24070904	24389670	318	17
11[#]	10867613	C:T	0.153	-	2.26E-05	3.48	10834263	10928827	94	3

Chr=chromosome; Pos= physical position of SNP; MAF=minor allele frequency; AE=allelic effect with reference to the minor allele (average traits value of genotypes carrying minor allele - average traits value of genotypes carrying major allele), LD= linkage disequilibrium. Known genes= total known genes observed within the LD block. PN=panicles per m^2; SS=seed set; TGW=thousand grain weight; SP= spikelets per m^2; HI=harvest index; TDW= total dry weight; NGDW=non-grain dry weight; PH=plant height; DTF=days to flowering.

Supplementary Table S7. Common genetic loci detected across treatments (non-stress [NS] vs. water-deficit stress [WD]) in 2013 or 2014 (**A**). Similarly, common genetic loci detected across years (2013 vs. 2014) in NS or WD conditions (**B**).

**(A) Common genetic loci detected across treatments in either 2013 or 2014**							
**2013**				**2014**			
**Chromosome**	**Position**	**NS**	**WD**	**Chromosome**	**Position**	**NS**	**WD**
7	22699138	SP	SP and SPP	1	38286772	PH	PH
4	23423399	SPP	SPP	3	72105	DTF	DTF
5	5366489	TGW	TGW	12	24162384	DTF	DTF
1	38286772	PH	PH	11	10867613	NGDW	NGDW
3	21686259/**21670338**	DTF	DTF	12	10443628/**10611754**	SP	SP
3	21686358/**21944343**	DTF	DTF				
3	5113428/**5113535**	DTF	DTF				
12	24162384	NGDW	NGDW				
4	34815309/**34815277**	TDW	TDW				
**(B) Common genetic loci detected across years in NS or WD conditions**							
		**2013**	**2014**			**2013**	**2014**
**Chromosome**	**Position**	**NS**		**Chromosome**	**Position**	**WD**	
10	19903199	PN	PN	3	16725803	TGW	TGW
4	23423399	SPP	SPP	1	38286772	PH	PH
2	30699332	TGW	TGW				
5	5366489	TGW	TGW				
1	38286772	PH	PH				
3	5113428	DTF	DTF				

Bold SNPs are detected in WD conditions and falling within the same linkage disequilibrium (LD) block. PN=panicles per m^2, SP=spikelets per m^2; SPP=spikelets per panicle; TGW=thousand grain weight; PH=plant height; DTF=days to flowering; NGDW=non-grain dry weight; TDW=total dry weight. The markers position in bold are different SNPs but fall within the same LD block

Supplementary Table S8: The details of genetic loci detected for corrected grain yield components and related traits (only on harvest index excluding the other traits in this group) in 2013 water-deficit stress conditions using compressed mixed linear-model (CMLM) and multi-locus mixed model (MLMM) methods. The chromosome loci detected through both the methods were marked by asterisk sign (*); those detected through only by MLMM were marked by hashtag (#) sign. The remaining all unmarked chromosome loci were detected only through CMLM method. Trait acronyms are given in Table 1.

Traits	Chr	Pos	Allele	MAF	*P* value$_{CMLM}$	*P* value$_{MLMM}$	AE	LD block		Size (kb)	Known genes
								Start	End		
**Grain yield component traits**											
PN	8*	20408464	G:T	0.052	1.39E-06	1.77E-06	44.65	20199466	20450490	251	14
	6*	9774102	C:T	0.151	7.57E-06	5.05E-11	28.01	9774102	9992897	218	10
	2	4668201	A:G	0.476	9.44E-05	-	-15.91	4668201	4711157	42	6
	7#	15365358	T:C	0.351	-	1.57E-06	-16.42	15293375	15388056	94	5
	4#	22006507	C:T	0.264	-	1.85E-05	15.47	21989957	22015135	25	3
	8#	27943348	G:A	0.052	-	1.25E-05	24.89	27905391	27943348	37	8
	4#	15853443	C:G	0.374	-	4.76E-05	14.60	15637881	15853443	215	2
SPP	4*	23471311	C:G	0.365	7.00E-06	3.09E-08	-8.96	23428565	23483270	54	5
	4	20027177	A:G	0.052	4.32E-05	-	14.81	20014494	20066427	51	4
	6*	9871701	A:G	0.144	6.33E-05	8.99E-06	-9.89	9774102	9992897	218	10
	3#	29663197	C:T	0.257	-	4.69E-06	-6.79	29614230	29742101	127	7
	1#	855970	G:C	0.167	-	1.85E-05	5.76	769982	931087	161	19
	11#	23178024	C:T	0.086	-	3.18E-05	8.37	23178024	23183705	5	1
SS	6*	9871701	A:G	0.144	1.37E-05	5.46E-09	-5.93	9774102	9992897	218	10
	11	17886595	C:T	0.297	7.11E-05	-	-3.64	17874496	18026910	152	2
	3*	5113428	T:C	0.424	8.63E-05	4.12E-08	-3.76	5021158	5167439	146	13
	6	10086748	C:G	0.188	9.81E-05	-	-4.26	10086745	10132707	45	4
	7#	21266079	C:T	0.096	-	6.95E-06	-3.49	21245869	21290877	45	3
	5#	29213653	C:A	0.140	-	2.96E-05	3.38	29213653	29238030	24	4
	12#	27244607	G:A	0.055	-	7.01E-05	-5.56	27093600	27244607	151	14
SP	12*	141599	G:A	0.122	2.66E-05	2.52E-05	-2.29	141599	148272	6	1
**Other related traits**											
HI	4*	34815277	C:T	0.074	1.98E-07	5.40E-06	-0.06	34815277	34833179	17	5
	3*	5113428	T:C	0.424	4.62E-06	1.03E-06	-0.03	5021158	5167439	146	13
	11	10627944	A:C	0.074	8.20E-06	-	-0.05	10627944	10863355	235	9
	11	10131062	T:C	0.055	3.20E-05	-	-0.06	9838715	10131062	292	3
	11	10329677	C:A	0.055	3.20E-05	-	-0.06	10265286	10341103	75	2
	6	10086748	C:G	0.188	5.88E-05	-	-0.03	10036641	10086748	50	1
	6	2950054	G:T	0.092	6.57E-05	-	-0.04	2888879	2981224	92	12
	1	604746	A:G	0.452	9.43E-05	-	-0.03	557715	717021	159	12

Chr=chromosome; Pos= physical position of SNP; MAF=minor allele frequency; AE=allelic effect with reference to the minor allele (average traits value of genotypes carrying minor allele - average traits value of genotypes carrying major allele). Known genes=total known genes observed within the LD block. PN=panicles per m^2; SPP=spikelets per panicle; SS=seed set; SP=spikelets per m^2; HI=harvest index.

Supplementary Table S9: The list of *a priori* candidate genes within the linkage disequilibrium block of GWAS significant peak SNP/loci for grain yield in non-stress (NS) and water-deficit stress (WD) conditions. Genes with bold annotation were responsive to abiotic stress stimulus (Gene Ontology [GO]:0009628).

Trt	Year	Locus Name	Distance to peak SNP	Gene ID	Gene annotation	Putative function	Reference
NS	2013	Q1	6	LOC_Os11g17970.1	Pot family protein also called as PTR2 peptide transport	ABA and nitrate transport; Rice homologue ***short panicle 1** (PTR2)* regulate panicle and grain size.	(Li *et al.*, 2009)
			31	LOC_Os11g18044.1	Peptide transporter PTR2		
		Q2	55	LOC_Os02g49850.1	Plastocyanin	Flower development (GO:0009908) and pollination (GO:0009856) in rice; *At* orthologue regulate anther development, seed set and pollen tube growth.	(Dong *et al.*, 2005)
	2014	Q3	Within	LOC_Os12g23310.1	Expressed protein	-	-
		Q4	34	LOC_Os03g47470.1	STE kinases include homologs to sterile 7, 11 and 20	*At* orthologue (*At*STE or BLUE LIGHT SIGNALING1 [BLUS1]) of this gene (AT4G14480.1) is a major stomatal regulator to enhance CO_2 assimilation.	(Takemiya *et al.*, 2013)
		Q5	48	LOC_Os02g47744.1	MYB family transcription factor	Cell growth and differentiation; response to biotic and abiotic stress	(Ambawat *et al.*, 2013)
		Q6	3	LOC_Os10g36229.1	**Mov34/MPN/PAD-1 family protein**	Multicellular organismal development; *AT* (AT5G56280.1) regulate COP9 signalosome assembly and photomorphogenesis.	-
		Q7	Within	LOC_Os12g09670.1	Expressed protein	-	-
		Q8	13	LOC_Os01g73580.1	Glycosyl hydrolases	Carbohydrate metabolic process; role in rice grain filling.	(Liu e*t al.*, 2010)
WD	2013	Q9	13	LOC_Os04g58580.1	**Phosphomannomutase**	Carbohydrate metabolic process; L-ascorbic acid biosynthesis that act as a redox buffer to detoxify reactive oxygen species.	(Arrigoni and De Tullio, 2002)
		Q10	Within	LOC_Os03g10100.1	Sugar transporter	Allocation of sugar between sinks; regulate water-deficit stress tolerance.	(Jarzyniak and Jasiński, 2014)

		5	LOC_Os03g10120.1	**COP9 signalosome complex subunit 4**	Arabidopsis flower development.	(Wang *et al.*, 2003)
2014	Q11	16	LOC_Os03g12900.1<br>LOC_Os03g12910.1	**Squalene monooxygenase**<br>**Squalene monooxygenase**	Regulates reactive oxygen species, stomatal responses and water-deficit stress tolerance.	(Posé *et al.*, 2009)
	Q12	6	LOC_Os01g72630.1	Expressed protein	-	-
	Q13	3	LOC_Os10g30790.2	Inorganic phosphate transporter	Response to phosphate starvation, altering root system architecture in response to phosphate starvation, response to heat.	(Miura *et al.*, 2011), (Pacak *et al.*, 2016)
	Q15	712*bp*	LOC_Os11g44810.1	Auxin-repressed protein	Induced under water-deficit stress.	(Govind *et al.*, 2009)

At: Arabidopsis thaliana

Supplementary Table S10: The details of genetic loci detected from previously published data on grain yield and yield components from Kikuchi et al. (2017), and number of spikelets per panicle (a key yield component) from Rebolledo et al. (2016), using the same rice PRAY panel. The GWAS analysis between phenotypic traits and genotypic markers (46K) was conducted using compressed mixed linear model (CMLM) and multi-locus mixed model (MLMM) methods. The loci detected by both the methods were marked by asterisk (*) sign; those detected through only by MLMM were marked by hashtag (#) sign. The remaining all unmarked loci were detected only through CMLM method. The genetic locus that was detected for grain yield on chromosome 2 (Q2: Pos=30523925) in 2013 non-stress conditions (for details see Table 3), was overlapped with the locus for panicle weight (Pos=30518548; different SNP but falls within same LD block of Q2 locus) under low planting density (highlighted bold in table).

Traits	Trt	Chr	Pos	Allele	MAF	P value$_{CMLM}$	P value$_{MLMM}$	AE
**(A) Rebolledo et al. (2016)**								
Number of spikelets per panicle (Experiment 1)	Normal irrigated condition	2*	22645474	A:G	0.080	7.09E-05	1.53E-07	16.37
		5	6678610	C:T	0.418	9.42E-05	-	9.48
		5#	28808160	T:C	0.160	-	4.94E-07	13.05
		9#	12078875	T:A	0.417	-	4.91E-05	9.66
		4#	3419364	G:A	0.142	-	7.41E-05	10.60
Number of spikelets per panicle (Experiment 2)		7*	22001703	G:A	0.066	3.30E-06	8.14E-07	14.97
		5*	28763902	G:A	0.164	1.00E-05	4.39E-06	10.40
		2#	7167677	A:G	0.014	-	1.21E-05	-7.51
		5#	5847976	C:T	0.311	-	9.91E-05	-5.28
**(B) Kikuchi et al. (2017)**								
Panicle weight ($g\ m^{-2}$)	Normal planting density	-	-	-	-	-	-	-
Panicle number (m^{-2})		-	-	-	-	-	-	-
Days to heading (or flowering)		7	3488686	C:G	0.323	4.85E-05	-	-3.74
		6*	9774102	C:T	0.156	4.88E-05	2.43E-05	-4.39
		6	9871701	A:G	0.153	6.71E-05	-	-4.34
		6#	7203734	T:G	0.249	-	1.71E-05	3.71
		7#	27496103	G:T	0.080	-	5.24E-05	4.47
Total biomass ($g\ m^{-2}$)		6*	2185018	T:C	0.113	6.67E-05	4.86E-05	-124.86
Harvest index		-	-	-	-	-	-	-
Panicle weight ($g\ m^{-2}$)	Low Planting density	6*	24259361	C:T	0.140	1.2580E-04	7.31E-05	-39.62
		1#	24856633	G:T	0.473	-	7.47E-06	-25.95
		**2#**	**30518548**	**G:C**	**0.099**	**-**	**3.71E-07**	**-50.79**
		3#	13026098	C:T	0.061	-	8.35E-05	-54.00
Panicle number (m^{-2})		-	-	-	-	-	-	-
Days to heading (or flowering)		7	3488686	C:G	0.323	4.31E-05	-	-3.77
		6*	9774102	C:T	0.156	6.44E-05	2.23E-06	-4.32
		6	9871701	A:G	0.153	8.73E-05	-	-4.26
		1#	855970	G:C	0.160	-	2.23E-06	3.61
		6#	7182493	G:A	0.221	-	1.41E-05	3.27
		4#	34314696	G:T	0.055	-	3.74E-05	-5.01
Total biomass ($g\ m^{-2}$)		-	-	-	-	-	-	-
Harvest index		11*	333550	T:C	0.177	1.94E-05	1.70E-08	0.04
		1*	852462	T:C	0.165	4.10E-05	8.75E-08	-0.04
		12#	14049048	C:T	0.181	8.86E-07	-	-0.03
		11#	3476816	T:C	0.106	5.63E-06	-	0.03

Yield and yield components data of Kikuchi et al. (2017) study was converted from per plant or hill to per m^2 area. Chr=chromosome, Pos=position of SNP, MAF=minor allele frequency, AE=allelic effect regarding the minor allele (average trait value of genotypes carrying minor allele- average traits value carrying the major allele).

References

Ambawat S, Sharma P, Yadav NR, Yadav RC. 2013. MYB transcription factor genes as regulators for plant responses: an overview. Physiology and Molecular Biology of Plants **19,** 307-321.

Arrigoni O, De Tullio MC. 2002. Ascorbic acid: much more than just an antioxidant. Biochimica et Biophysica Acta (BBA) - General Subjects **1569,** 1-9.

Dong J, Kim ST, Lord EM. 2005. Plantacyanin plays a role in reproduction in Arabidopsis. Plant Physiology **138,** 778-789.

Govind G, Vokkaliga Thammegowda H, Jayaker Kalaiarasi P, Iyer DR, Muthappa SK, Nese S, Makarla UK. 2009 Identification and functional validation of a unique set of drought induced genes preferentially expressed in response to gradual water stress in peanut. Molecular Genetics and Genomics **281,** 591-605.

Jarzyniak KM, Jasiński M. 2014. Membrane transporters and drought resistance – a complex issue. Frontiers in Plant Science **5,** 687.

Kikuchi S, Bheemanahalli R, Jagadish KSV, Kumagai E, Masuya Y, Kuroda E, Raghavan C, Dingkuhn M, Abe A, Shimono H. 2017. Genome-wide association mapping for phenotypic plasticity in rice. Plant, Cell & Environment **40,** 1565-1575.

Li S, Qian Q, Fu Z, Zeng D, Meng X, Kyozuka J, Maekawa M, Zhu X, Zhang J, Li J, Wang Y. 2009. *Short panicle1* encodes a putative PTR family transporter and determines rice panicle size. The Plant Journal **58,** 592-605.

Liu X, Guo T, Wan X, Wang H, Zhu M, Li A, Su N, Shen Y, Mao B, Zhai H, Mao L, Wan J. 2010. Transcriptome analysis of grain-filling caryopses reveals involvement of multiple regulatory pathways in chalky grain formation in rice. BMC Genomics **11,** 730.

Miura K, Lee J, Gong Q, Ma S, Jin JB, Yoo CY, Miura T, Sato A, Bohnert HJ, Hasegawa PM. 2011. SIZ1 Regulation of Phosphate Starvation-Induced Root Architecture Remodeling Involves the Control of Auxin Accumulation. Plant Physiology **155,** 1000-1012.

Pacak A, Barciszewska-Pacak M, Swida-Barteczka A, Kruszka K, Sega P, Milanowska K, Jakobsen I, Jarmolowski A, Szweykowska-Kulinska Z. 2016. Heat Stress Affects Pi-related Genes Expression and Inorganic Phosphate Deposition/Accumulation in Barley. Frontiers in Plant Science **7,** 926.

Posé D, Castanedo I, Borsani O, Nieto B, Rosado A, Taconnat L, Ferrer A, Dolan L, Valpuesta V, Botella MA. 2009. Identification of the Arabidopsis dry2/sqe1-5 mutant reveals a central role for sterols in drought tolerance and regulation of reactive oxygen species. The Plant Journal **59,** 63-76.

Rebolledo MC, Peña AL, Duitama J, Cruz DF, Dingkuhn M, Grenier C, Tohme J. 2016. Combining image analysis, genome wide association studies and different field trials to reveal stable genetic regions related to panicle architecture and the number of spikelets per panicle in rice. Frontiers in Plant Science **7,** 1384.

Takemiya A, Sugiyama N, Fujimoto H, Tsutsumi T, Yamauchi S, Hiyama A, Tada Y, Christie JM, Shimazaki KI. 2013. Phosphorylation of BLUS1 kinase by phototropins is a primary step in stomatal opening. Nature Communications **4,** 2094.

Wang X, Feng S, Nakayama N, Crosby WL, Irish V, Deng XW, Wei N. 2003. The COP9 signalosome interacts with SCF(UFO) and participates in Arabidopsis flower development. The Plant Cell **15,** 1071-1082.
